# Gene expression networks regulated by human personality

**DOI:** 10.1038/s41380-024-02484-x

**Published:** 2024-03-04

**Authors:** Coral del Val, Elisa Díaz de la Guardia-Bolívar, Igor Zwir, Pashupati P. Mishra, Alberto Mesa, Ramiro Salas, Guillermo F. Poblete, Gabriel de Erausquin, Emma Raitoharju, Mika Kähönen, Olli Raitakari, Liisa Keltikangas-Järvinen, Terho Lehtimäki, Claude Robert Cloninger

**Affiliations:** 1 University of Granada, Department of Computer Science and Artificial Intelligence, Andalusian Research Institute in Data Science and Computational Intelligence, Granada, Spain; 2 Instituto de Investigación Biosanitaria de Granada (ibs. GRANADA), Granada, Spain; 3 Washington University School of Medicine, Department of Psychiatry, St. Louis, MO USA; 4 Tampere University, Department of Clinical Chemistry, Fimlab Laboratories, and Finnish Cardiovascular Research Center – Tampere, Faculty of Medicine and Health Technology, Tampere, Finland; 5 The Menninger Clinic, Baylor College of Medicine, and DeBakey VA Medical Center, Houston, TX USA; 6 The Menninger Clinic, Houston, TX USA; 7 University of Texas Health San Antonio, Long School of Medicine, Department of Neurology, Biggs Institute of Alzheimer’s & Neurodegenerative Disorders, San Antonio, TX USA; 8 Department of Clinical Physiology, Tampere University Hospital, and Finnish Cardiovascular Research Center - Tampere, Faculty of Medicine and Health Technology, Tampere University, Tampere, Finland; 9 University of Turku and Turku University Hospital, Center for Population Health Research; University of Turku, Research Center of Applied and Preventive Cardiovascular Medicine; Turku University Hospital, Department of Clinical Physiology and Nuclear Medicine, Turku, Finland; 10 University of Helsinki, Department of Psychology and Logopedics, Helsinki, Finland

**Keywords:** Genetics, Psychology, Biological techniques

## Abstract

Genome-wide association studies of human personality have been carried out, but transcription of the whole genome has not been studied in relation to personality in humans. We collected genome-wide expression profiles of adults to characterize the regulation of expression and function in genes related to human personality. We devised an innovative multi-omic approach to network analysis to identify the key control elements and interactions in multi-modular networks. We identified sets of transcribed genes that were co-expressed in specific brain regions with genes known to be associated with personality. Then we identified the minimum networks for the co-localized genes using bioinformatic resources. Subjects were 459 adults from the Young Finns Study who completed the Temperament and Character Inventory and provided peripheral blood for genomic and transcriptomic analysis. We identified an extrinsic network of 45 regulatory genes from seed genes in brain regions involved in self-regulation of emotional reactivity to extracellular stimuli (e.g., self-regulation of anxiety) and an intrinsic network of 43 regulatory genes from seed genes in brain regions involved in self-regulation of interpretations of meaning (e.g., production of concepts and language). We discovered that interactions between the two networks were coordinated by a control hub of 3 miRNAs and 3 protein-coding genes shared by both. Interactions of the control hub with proteins and ncRNAs identified more than 100 genes that overlap directly with known personality-related genes and more than another 4000 genes that interact indirectly. We conclude that the six-gene hub is the crux of an integrative network that orchestrates information-transfer throughout a multi-modular system of over 4000 genes enriched in liquid-liquid-phase-separation (LLPS)-related RNAs, diverse transcription factors, and hominid-specific miRNAs and lncRNAs. Gene expression networks associated with human personality regulate neuronal plasticity, epigenesis, and adaptive functioning by the interactions of salience and meaning in self-awareness.

## Introduction

### The complexity of gene interactions and gene expression

There is a growing body of evidence that genes do not operate in isolation, but rather form vast and complex information-processing networks of interacting genes, proteins, and small molecules [1–3]. In humans, these networks are self-organized as specialized functional modules that interact collaboratively by turning one another on and off to adapt to changing external and internal conditions [4, 5]. Such reciprocal feedback interactions coordinate information-transfer [6], thereby promoting a person’s healthy development and longevity despite stressors [7, 8].

For example, apoptosis is a form of programmed cell death that occurs in multicellular organisms and plays an important role in development and control of tissue homeostasis by protecting cells against threatening stimuli. Cell death is executed through two core interactive pathways, an extrinsic (death receptor-mediated) pathway and an intrinsic (mitochondria-mediated) pathway [9–11]. These processes are tightly controlled by positive and negative regulators that activate or inhibit death receptor signaling [9–13]. One of these regulators is the Fas-Apoptotic Inhibitory Molecule (FAIM), which has an important role in neuronal processes, including neurotrophin-induced stimulation of neurite outgrowth and synaptic transmission, in addition to its role as a death receptor antagonist [9, 14]. The microRNA miR-1-3p directly regulates FAIM pathways [9]. Furthermore, microRNAs (miRNAs) and neurotrophins regulate each other, thereby integrating the positive and negative interactions in their signaling pathways [14].

Such interactive modules bring together many diverse, and frequently multifunctional, molecules that enable new emergent functions when they operate jointly [15]. In turn, different modules with distinct functions can interact with one another to form coordinated information-processing networks that enable integrated responses to changing conditions in ways that are flexible, efficient, collaborative, and open-ended (i.e., unpredictable and creative expression of potential with no determined limit, as is prominent in evolution) [6, 16]. Consequently, the complex adaptive functions of biological systems depend upon both their specific constituent molecules and the information encrypted in the organization of dynamic multi-modular networks, as is prominent for learning in the brain [4, 17].

As a result of the crucial role of information encrypted within complex networks, independent variation in a single gene is rarely necessary or sufficient to cause or protect against disease [5, 18, 19]. Even in rare instances where a single gene is a sufficient cause or a major risk factor for disease, its phenotypic expression is modulated by interactions among multiple other genes and environmental factors [19, 20]. For example, the TP53 gene encodes a 393-amino-acid protein called p53, which has a key role in the intrinsic apoptosis pathway and acts as a tumor suppressor because of its position within a network of transcription factors (TFs) [4, 21]. The TP53 gene is mutated in about half of human cancers. It has been nicknamed the “guardian of the genome” because it prevents the proliferation of cells with damaged DNA and thereby maintains genome integrity [21].

In response to different cellular stressors, p53 activates the transcription of selective targets needed for various forms of cellular stress, such as DNA repair, cell cycle arrest, apoptosis, or senescence [22]. These selective functions are properties of the p53 protein itself, but neither p53 nor its network can function as a tumor suppressor in isolation [4]. Thus, both interactive sets of genes and integrated sets of interactive biological networks have been characterized as symbiotic systems that are favorable because they increase fitness in combination [4]. Such symbiotic network properties extend beyond individual organisms in all life domains to social and ecological communities and to the planetary biosphere, creating a nested hierarchy of complex biological systems [23–26].

### Sources of evidence of the complex regulation of gene expression

The recognition and growing understanding of the crucial role of complex functional networks, rather than independent genes, has come from several lines of evidence targeting different steps in gene expression. Gene expression is a complex process involving coordination of dynamic events, which are subject to regulation and integration across multiple levels, each with multiple steps: the transcriptional level from DNA to RNA (i.e., transcription initiation, elongation, and termination), the posttranscriptional level (i.e., RNA translocation, splicing, and stability), the translational level from RNAs to proteins (translation initiation, elongation, and termination), and the posttranslational level (protein splicing, translocation, stability, and covalent modifications) [27]. TFs that bind to genomic DNA and recruit or bind proteins, as well as the regulation of epigenetic modification of chromatin and its constituent DNA, RNA, and proteins, are the major influences on transcriptional regulation [27, 28]. MiRNA-gene interactions are the major influences after transcriptional regulation [27, 28].

Genome-wide profiling of gene expression using RNA microarray or sequence analysis has provided tools that are useful to identify the intricate patterns of interactions between multiple types of genes and the environment [29]. Gene expression profiling studies involve measuring the expression of thousands of genes in an organism at the same time. These studies have shown that genes are often co-expressed in patterns, suggesting that they are components of a functional network. These complex interactions regulate both gene expression levels and the epigenetic changes that influence health and disease [19].

Direct evidence that genes, RNA transcripts, proteins, and other molecules function in complex networks has come from studies of each component of regulatory processing in gene expression. For example, gene knockout experiments involve knocking out or deleting a single gene in an organism. When a gene is deleted, it can have a dramatic effect on the expression of other genes that interact with it in a functional network [30]. Gene knockout experiments provide direct evidence of modular interactive networks, but are limited to experimental animals that fail to reflect the complexity of the human brain [31, 32] or emotional, cognitive, and social features that require self-awareness [31–33] (Supplementary Text S1).

There are about 63,000 genes in the human genome, all composed of DNA; 20,000 of these are transcribed to RNAs that encode proteins, whereas the other 43,000 are transcribed to non-protein-coding RNAs (ncRNAs) that serve a variety of regulatory functions [7, 34, 35].

In the first regulatory step of gene expression, TFs interpret the information encrypted in the genomic code by regulating the transcription of DNA sequences to RNA sequences. TFs are proteins that recognize and bind to specific DNA sequences to control chromatin and transcription [36, 37]. Each TF in humans binds to an average of more than 100 different genes, suggesting that TF-gene interactions form complex functional networks [37, 38]. TFs also bind or recruit other types of proteins with which they collaborate in transcription regulation; these proteins include chromatin remodelers, cofactors, and transcription initiation factors that help to coordinate the spatiotemporal details of gene expression [38]. TFs even link apparently unrelated functional processes for an emergent function that arises when they are coordinated in an interactive network, even when these different processes do not share common genes [38].

Alternative splicing of the 20,000 protein-coding genes in the human genome produces multiple mRNAs for each gene, resulting in production of an average of 4 or more proteins per gene. The 80 to 100 thousand cellular proteins that are produced by alternative splicing in various contexts interact with one another in many functional networks, rather than operating independently [39]. Many single-nucleotide polymorphisms (SNPs) associated with individual differences in health and disease occur in or near protein-protein interfaces [35, 40]. Such SNPs often affect later post-translational modifications of proteins, such as phosphorylation, ubiquitination, methylation, acetylation, and glycosylation, which influence risk of complex traits and disorders [41]. Consequently, a thorough understanding of the regulation of gene expression must consider the collaborative relationships of genes with protein-protein interaction networks because it is the regulated activities of various kinds of proteins, including enzymes, receptors, and TFs, that influence phenotypes and their development [39, 42, 43].

After DNA variants in the genome have been transcribed to RNAs (protein-coding or non-coding regulatory variants), non-coding RNA (ncRNA) variants have been shown to exert strong regulatory influences on gene expression rates and localization of co-expression. At the post-transcriptional level, miRNAs have been found to interact with long-non-coding RNAs (lncRNAs) and other genes in large functional networks in all life forms, with substantial conservations and differences among the domains of life and in phylogenesis [27, 44–46]. MiRNAs are small ncRNAs that regulate gene expression through mRNA destabilization and translational repression [47, 48]. They play a major role in healthy human brain development and in the brain-related pathogenesis of developmental, degenerative, and psychiatric disorders [49].

Nearly half of human miRNAs are expressed in the brain where they regulate basic neural processes, including neurogenesis and neuroplasticity [50]. These miRNAs are crucial components of the gene networks that regulate adult neurogenesis, which is important for learning and memory [51]. MiRNAs also work in coordination with lncRNAs to regulate which genes are co-expressed in the same cells or differentiated functional regions [7].

### The post-genomic revolution: focus on regulation of gene expression networks

After the genomes of humans and many other organisms were nearly fully sequenced, the importance of the regulation of gene expression and epigenetic change was recognized. There was a paradigm shift in how to understand the evolution and development of phenotypes in health and disease at the beginning of the 21st century. For example, when the first miRNA gene was discovered in 1993, it was considered an unusual way to control developmental timing in nematodes [52, 53]. However, by 2000 it was recognized that animals had evolved many miRNAs that organized networks of interactive genes, which in turn enabled greater structural and functional complexity than is found in plants [44, 52].

In addition, the emergence of network science in the 21^st^ century provided the mathematical tools to characterize the structure and dynamic control of complex systems for the first time [54–56]. Network scientists recognized that the increasing modularity of gene expression networks enabled efficient information-processing that optimized the trade-off in complex networks between topological complexity (i.e., coordinated multi-regional expression and functional collaboration) and the costs of increased structural connectivity [57].

For example, information processing has been shown to follow principles of modular design that are fractal-like in the human brain with localized gene co-expression in different brain regions [57], which are homologous to the organization of metabolic networks at scales from individuals to the whole biosphere [25]. These observations have required molecular geneticists to “reorganize [their] view of the universe” [44, 52] to accept a paradigm in which gene expression is regulated by complex information-processing networks that are dynamically adaptive [27, 58], efficiently self-organized in interactive modules [59], and collaboratively turn one another on and off to respond to changing conditions in a context-dependent manner that is open-ended [6, 9, 60].

This dramatic change in perspective has been called the “post-genomic revolution” [19]. Rather than thinking in terms of one-to-one relationships between a single gene in the genome and its functions, as was done in early genomic studies, it is now widely recognized that the information stored in DNA is encrypted in a fashion so that it can lead to different sequences of events under different environmental conditions by means of differential regulation of gene expression and epigenetic change. Put another way, to decode the information encrypted in the genome requires consideration of the processes by which gene expression and epigenetic change are choreographed in developmental sequences and regulated by complex multi-modular networks under changing conditions.

### Increased level of consciousness in human evolution

The emergence of self-awareness in modern *Homo sapiens* is itself a radical shift in internal and external regulatory conditions [7, 8, 18, 61–66]. How did humans become able to self-regulate their own gene expression, biopsychosocial development, and health? We have addressed these fundamental and challenging questions by studying human personality, which is defined as the way a person learns both to shape and to adapt to ever-changing external and internal events [63, 65]. We identified the three systems of learning and memory underlying human personality, which evolved by incremental stages in evolution: associative conditioning of emotional reactivity and habits, intentional self-control, and creative self-awareness [7, 64, 65]. We have been able to distinguish these three systems in terms of their personality-based phenotypic organization and genotypic-environmental interaction networks [64], developmental dynamics in relation to physical, mental, social, and spiritual well-being [61, 62, 66–68], social values [66, 69, 70], and brain functional connectivity [8].

We have replicated the phenotypic and genotypic findings in multiple countries (Finland, Germany, South Korea) with different cultural and environmental conditions [64], and replicated the phenotypic organization of personality in relation to neuropsychological functioning and well-being in countries on all continents around the world [67–69, 71–77].

The dynamics of personality development is complex with feedback interactions among many biogenetic, psychosocial, environmental influences, and sociocultural influences [63, 74, 78]. Consequently, its development is meta-stable and saltatory; that is, personality development occurs abruptly in jumps to higher or lower levels of well-being at the extremes of tipping points due to changes in a person’s internal and external conditions [63, 79, 80]. The character components change with shifts in awareness that either enlarge or reduce consciousness [69, 80, 81]. Therefore, personality development has multiple stages with incremental shifts in maturity and integration [71]. However, a person’s identity (i.e., continuity in time of characteristic interests, habits, goals, and values) is relatively stable in young adults. Consequently, the test-retest correlations between personality ratings stay around 0.8 over both months and decades among adults 30–50 years of age in the general population, as in the Young Finns Study [82].

An individual’s sense of identity and joint temperament-character configuration remains stable over long periods of time, and personality is a strong predictor of a person’s overall level of well-being and their overall burden of disease [83]. Accordingly, it is important to understand how integrated configurations of character and temperament in adults, which are usually trait-like (i.e., stable from day to day and year to year), can coordinate how a person self-regulates their functioning under changing conditions? To answer this question, we carried out a multi-omic study of specific testable hypotheses.

### Hypotheses to be tested with integrated genomic-transcriptomic data

Phenotypic evolution is linked to the evolution of the regulatory systems for gene expression [27], so we predict differential gene expression according to a person’s character-temperament profile as an indicator of their level of self-regulatory capacity promoting well-being (i.e., creative > organized > unregulated) [7, 64]. Furthermore, functional interactions of genes that promote fitness and emergent adaptive functions confer a natural selective advantage, so they tend to be conserved in evolution [3, 84]. Therefore, we formulated testable predictions about the molecular mechanisms by which gene expression networks have evolved incrementally to promote selective advantage by emergent functions of increasing plasticity, complexity, and consciousness. We predict that the constituents of specialized functional modules are enriched in the following key types of molecules based on prior research on the evolution of gene expression networks in the ancestral lineage of modern human beings [66, 70]:

First, RNAs related to the process of *liquid-liquid phase separation* (LLPS) are hypothesized to be fundamental in the origin of cellular life and the organization of specialized intracellular functions [85–87]. Here we test whether LLPS-related RNAs have been conserved in evolution to form membrane-less organelles as compartments for synthesis within cells, such as stress granules in which gene expression is reversibly adapted to stressful conditions [88–90].

Second, genes related to *TF binding* to DNA and the recruitment and binding of other proteins are fundamental for the formation of protein-protein interaction networks [27, 28, 91]. The evolution of multicellular organisms is hypothesized to depend on the evolution of complex transcriptional regulatory networks to control cellular differentiation, especially in plants and animals, due to their orchestrated embryonic development [92]. However, few new families of TFs have arisen since the divergence of plants and animals from a common unicellular organism [27, 93].

Third, *gene-miRNA interactions* are hypothesized to be key mechanisms for post-transcriptional regulation of gene expression [27, 28, 44, 58]. Animals have evolved much greater numbers and diversity of miRNAs than plants and other life forms [27, 44]. The evolution of miRNAs is hypothesized to be key for the evolution of regulatory processes associated with increasing neuronal plasticity, complexity, and consciousness in bilaterians (i.e., animals with bilateral symmetry as an embryo), particularly in the lineage of primates and hominids, which have exceptionally high numbers and diversity of miRNAs unique to them [27, 44, 46, 94]. It is estimated that there are more than 2000 human miRNAs, each able to target hundreds of genes [27, 95]. Many miRNAs and lncRNAs expressed in the human brain are not conserved in chimpanzees [7, 94].

Fourth, there was incremental evolution of brain systems underlying personality, learning, and consciousness coincident with the increasing *miRNA and lncRNA diversity* in animals [7, 96, 97]. The highest level of central integration of information processing in the brains of vertebrates rises from the brainstem in fish to the hypothalamus in reptiles, neocortex in mammals, and then to the most recently evolved regions of the neocortex of modern human brains where the meaning of polymodal information is interpreted coherently in global context [66, 70]. We hypothesize that the evolution of self-awareness and related personality configurations required a unified and meta-stable perspective, as provided by human personality and its underlying resting-state functional brain connectivity [7, 66].

To test these hypotheses, we had access to phenomic, genomic, and transcriptomic data from a systematic epidemiological birth-cohort study in which individuals were young adults. Nevertheless, characterizing the regulation of gene expression and epigenetic modification in human adults presents many challenges for complex phenomena such as personality. Paradoxically, the challenges provide an especially strong test of our hypotheses, which is useful regardless of the outcome.

### Challenges in testing human genome-transcriptome relations

It is difficult to obtain relevant transcriptomic data about neuropsychiatric phenotypes such as human personality and brain functional connectivity. Unfortunately, transcripts from highly differentiated peripheral tissues, such as blood or saliva, may not be representative of transcripts from regions of interest, such as the brain when studying personality and related systems of learning and memory [32]. There have been suggestions that the transcriptome from assays of peripheral whole blood or leukocytes might be correlated with the transcriptome from brain regions of autopsied primates, but the overlap is minimal for complex phenotypes like human personality and regionally distributed brain networks [32, 98]. Specifically, genes that are expressed ubiquitously in both blood and most brain regions are often housekeeping genes involved in basic cellular processes, such as protein and mRNA metabolism, ribosomal activity, energy release, and cytoskeletal regulation [98]. In contrast, genes that are involved in many specific brain regions are rarely expressed in the blood of vervet monkeys [98]. Likewise, the expression of miRNAs in many brain regions of baboons showed limited overlap with expression in blood monocytes; even among 362 miRNAs widely expressed in the brain, only 11% were moderately correlated with the expression levels in blood monocytes [32].

In addition, post-mortem studies are complicated by small and selective samples and confounding variables, such as cause of death, postmortem interval, tissue pH, and RNA integrity [99]. Derived neuronal cell lines and brain organoids from pluripotent stem cells can allow the investigation of samples that are representative of brain tissue [100], but the development of these models is costly and their relationship to regulatory processes in naturally occurring miRNA expression networks in a self-aware person remains unknown [32].

Whereas genomic information is stable except for epigenetic modifications, a single sample of whole blood cannot provide information on all the transcriptomic processes that have occurred in each person’s life from embryogenesis through adulthood in the development and epigenesis of the brain and personality.

Although highly variable genes in the transcriptome of one whole blood sample are expected to have little or no direct overlap with personality-related SNPs from the genome, it may still be possible to identify the pathways and interaction networks by which personality-related genes identified in genome-wide association studies (GWAS) overlap with transcribed genes from even a single sample. Fortunately, extensive bioinformatic information is available about the interactive networks involved in regulating gene expression across a person’s entire lifespan.

### An innovative approach to overcoming the challenges

In view of the essential importance of transcriptomic studies for understanding regulatory networks for gene expression, we developed an innovative approach to overcome the anticipated difficulties with a phenotype as complex as human personality. Capitalizing on our prior replicated findings about personality-related genes related to personality from GWAS, we targeted genes that are co-expressed in the same brain regions as the known 972 personality-related genes from GWAS [64, 65]. We had already successfully identified such colocalized genes from GWAS to neuroimage the brain regions for the learning systems underlying human personality [7, 101]. Conservation of co-localized gene pairs implies a selective advantage, and therefore the genes are functionally related [3]. In the current project, we recognized that transcribed RNAs in whole blood were most likely to be functionally related to human personality if they were co-expressed in the same brain regions as personality-related genes from GWAS. After the colocalized genes and their interaction networks were identified, we could test this hypothesis based on the functional annotation of the brain regions and the colocalized genes.

Once the colocalized sets of transcribed genes and known personality-related genes were identified, we carried out network analysis to identify the key control elements and interactions in gene regulatory networks. The co-localized genes were used as seeds to identify the non-seed genes required to maintain network connectivity using bioinformatic resources about their known gene-gene interactions relevant to their whole lifespan, not just at one point in time. This allowed us to identify individual networks for colocalized genes in individuals with specific personality profiles associated with differentially regulated functional modules relevant to the dynamic orchestration of their whole life. Finally, we then examined the overlap and interactions among multiple specialized functional modules to identify their key control elements.

There are already multiple lines of evidence that support the relevance of the different specific classes of transcribed genes as considered in our hypotheses (Supplementary Text S1). However, this is the first study to identify the key control elements and specific functional modules in the networks by which gene expression is differentially regulated in multi-regional brain networks associated with distinct human personality profiles.

## Methods and Materials

### Original sample data and ethical approval

The study sample consisted of 459 young adults selected from the Young Finns Study (YFS), an epidemiological study of healthy Finnish children in birth-cohorts followed since 1980 when they were aged 3–18 years [102]. All subjects had thorough standardized genotypic and phenotypic assessments, including administration of the Temperament and Character Inventory (TCI) in 1997, 2001, 2007, and 2012 [61, 62]. Transcriptomic samples for 459 subjects (aged 34–49, women 54%) were processed by July 2013. The 459 subjects had provided blood for both genomic and transcriptomic assays and had prototypic TCI profiles previously assigned (i.e., creative-reliable, organized-reliable, and emotionally unregulated profiles [64]).

The YFS study received ethical approval from the Hospital District of Southwest Finland’s ethical committee on June 20, 2017 (ETMK: 68/1801/2017). All participants provided written informed consent and the study was conducted in compliance with the Declaration of Helsinki. Data privacy will be maintained in accordance with current regulations.

### RNA isolation

Whole blood of 2.5 ml was collected using PaXgene Blood RNA Tubes (PreAnalytix, Hombrechtikon, Switzerland) by inverting the tube 8–10 times and storing it at room temperature for a minimum of 2 h. The tubes were then frozen and stored for less than a year at a temperature of −80 °C. Upon thawing, the tubes were kept at room temperature for 2–12 h as per the instructions from PreAnalytix. RNA was extracted from the samples using the PAXgene Blood RNA Kit (Qiagen) along with the DNase Set, following the manufacturer’s instructions, and using the QiaCube device.

### RNA quality control

The concentration and quality of the RNA samples were assessed using a spectrophotometer, NanoDrop (BioPhotomer, Eppendorf, Wesseling-Berzdorf, Germany). The purity of the samples was determined based on the 260/280 ratio, which should be between 1.8 and 2.2. The validity of the RNA extraction process was confirmed by analyzing the RNA integrity using the RNA 6000 Nano Chip Kit (Agilent). The RNA integrity number (RIN) and the shape of the electropherogram were evaluated for 26 RNA samples obtained from YFS. Upon visual inspection, no signs of degradation were observed in any of the samples. The average RIN value was 8.2 with a standard deviation of 0.5. Additionally, the Agilent Small RNA Kit showed that the samples contained a small RNA fraction.

### RNA expression analysis

The expression levels were analyzed using the Illumina HumanHT-12 version 4 Expression BeadChip, which contains microarrays of 47,231 expression and 770 control probes. The process involved reverse-transcribing 200 ng of RNA into cDNA and labeling it with biotin-UTP using the Illumina TotalPrep RNA Amplification Kit (Ambion). 1500 ng of the resulting cDNA was then hybridized to the Illumina HumanHT-12 v4 Expression BeadChip and scanned with the Illumina iScan system. The raw data was exported from Beadstudio and processed in R (http://www.r-project.org/) using Bioconductor (http://www.bioconductor.org/). Nonparametric background correction was applied, followed by quantile normalization, log2 transformation and normalization of control and expression probes, using the neqc function in the limma package.

**Fig. 1:**
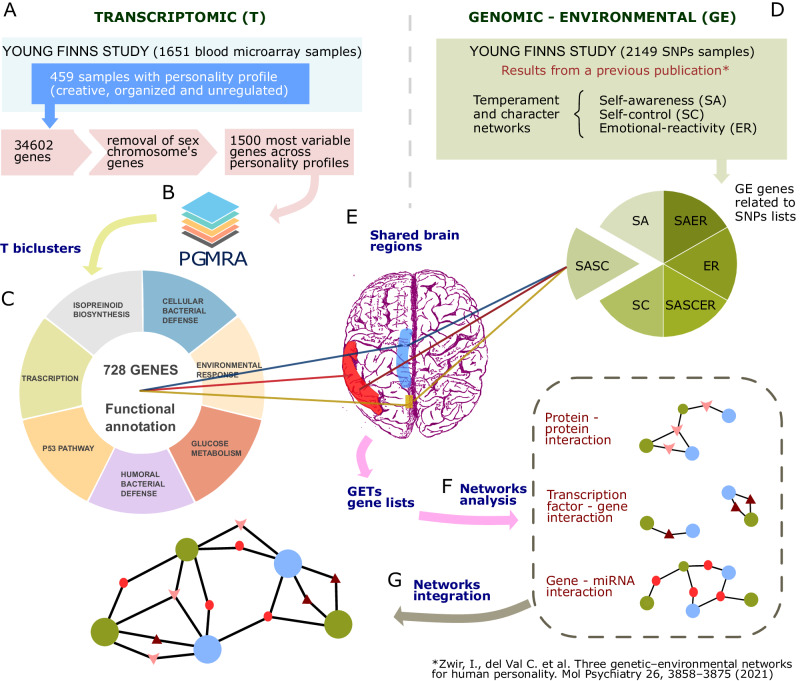
Methodology flowchart. **A** Transcriptomic (T) data processing workflow. **B** PGMRA analysis on T data and T- biclusters generation. **C** Functional annotation of PGMRA T-biclusters. **D** Collection of gene sets from the three Genomic-Environmental networks (GE) of human personality from Zwir et al. 2021. **E** Mapping of the T-biclusters and GE gene sets to brain regions, specifying GET gene subsets. **F** GET biological interaction analysis with three types of interaction networks (protein-protein, transcription factors-genes and gene-miRNA) *. **G** Integration of interactions networks for each GET *. *Node colors distinguish transcriptomic genes (blue), genomic-environmental genes (green), and newly introduced genes by the type of bioinformatic network analysis (red). Node shape indicates the molecular subtype for the new genes from network analysis: triangle (protein), star (TF), and circle (miRNA).

The expression data of 34,602 genes in blood samples from 459 individuals with a prototypic personality profile were further analyzed. The sex chromosome genes were removed, and the 1500 most variable genes across the three personality profiles were selected (Fig. 1A). For clarity, the seven steps in the analysis of the gene expression of these variably transcribed genes are depicted sequentially in a methodology flowchart (Fig. 1A–G) for the readers’ convenience.

### Identification of transcriptomics biclusters

The gene expression of those 1500 genes was analyzed using PGMRA [103], a data-driven, unsupervised machine learning method that incorporates the Generalized Factorization Method (GFM) and the Fuzzy Nonnegative Matrix Factorization (FNMF) algorithm [104–107]. PGMRA identifies naturally occurring sets of subjects with specific personality profiles that share the expression of certain genes. The subjects’ personality profiles were previously uncovered by PGMRA [64], whereas to create the transcriptomic subsets PGMRA was used as a feature selection method with a fixed number of clusters (*k* = 7), designated as Transcriptomic biclusters (T) (Fig. 1B). The functions of the transcriptomics biclusters’ genes were determined using GeneMANIA (https://genemania.org/) [108] (Fig. 1C. For two groups of genes, results could not be obtained, so they were searched using PANTHER (http://www.pantherdb.org/) [109].

### Linking genes from transcriptomic biclusters to brain regions

The genes within each PGMRA transcriptomic bicluster could be linked to various brain regions using the program Process Genes List (PGL) [101]. This machine learning technique calculates the average mRNA expression level for a gene list in each brain region, normalized using the Allen Brain Atlas. The brain regions in which these variably transcribed genes were significantly expressed were identified, as described previous work on the co-expression of the genes associated with the joint temperament-character networks [7, 65] (Fig. 1D, E).

### Linking the three personality-related networks’ genes to brain regions

In prior work we found that joint temperament-character configurations distinguish individuals in which one of three dissociable learning systems are predominant: emotional reactivity (ER), self-control (SC), and self-awareness (SA) [64]. These networks have nearly disjoint sets of genetic and environmental antecedents and distinctive effects on physical, mental, and social well-being. The phenotypic networks were strongly correlated with corresponding networks identified by genomic and/or environmental variables, so we refer to them as “genomic-environmental networks”. We identified the constituent genes of the genomic-environmental (GE) networks of human personality, including the Self-Awareness (SA), Self-Control (SC), and Emotional-Reactivity (ER) networks and their interactions when combined (SA/SC, SA/ER, SA/SC/ER) (Fig. 1D) Then we used PGL to determine the brain regions in which these genes exhibited significant co-expression with the variably transcribed genes (Fig. 1E).

### Analysis of Genomic-Environmental-Transcriptomic subsets (GETs)

The previous analysis allowed us to establish connections between the transcriptomic genes (i.e., “T genes” from the biclusters derived from gene expression data) and the genes of the genomic-environmental networks associated with human personality (“GE genes” from biclusters derived from genomic data), resulting in the creation of what we refer to as Genomic-Environmental-Transcriptomic (GET) subsets (Fig. 1F).

These GET subsets were analyzed using NetworkAnalyst (https://www.networkanalyst.ca/) [110]. For each GET subset, three types of biological interaction networks were created: protein-protein interactions (using the first-order IMEx Interactome database), gene-miRNA interactions (using the miRTarBase v8.0 database), and TF-gene interactions (using the ENCODE database) (Fig. 1F). The resulting networks were further processed by retaining only the minimum network (that is, the seed genes and essential non-seed genes that maintained network connectivity) (Fig. 1G). Each of the interaction networks for each GET subset was then downloaded, imported into Cytoscape software (version 3.9.1) for visualization [111], and combined into a single network (Fig. 1G). Tissue-specific values for the nervous system were added to the result using the STRINGify app within Cytoscape. When possible, the resulting networks were integrated together.

### Generation of an integrated GET network of personality

The shared genes between the selected GETs facilitated their integration in a single coordinated network. The single network integrated the transcriptomic (T) variants with the GET genomic subsets (SAER-SASC), so it can be designated as the T-SAER-SASC network. We used “all GET genes” as the query in NetworkAnalyst to find the missing genes connecting both GETs. The functional annotation of the resulting single coordinated network was performed using custom scripts to search the PubMed database (https://www.ncbi.nlm.nih.gov/pubmed/). The search terms for each gene included: [gene symbol], [neural AND gene symbol], [neuronal AND gene symbol], and [brain AND gene symbol]. The retrieved articles were manually inspected, and the functions of the genes were categorized based on known functions and related diseases, and then curated results were stored.

### Uncovering the Temperament-Character Molecular Integration Network (TCMIN)

The resulting single (T-SAER-SASC) network had a central regulatory hub of 6 shared genes. To infer the whole temperament-character molecular integration network (TCMIN) in which they were involved, we queried “all gene-miRNAs” in the database miRTarBase (v8.0) and protein-protein interactions from the IMEX interactome using NetworkAnalyst. Due to the large size of the resulting network, this had to be analyzed and visualized using various layout algorithms in Gephi (version 0.10.1), an open-source software that supports further network qualitative investigation. Network analysis and node overview, such as degree centrality, clustering coefficient, eigenvector centrality and modularity, were calculated to identify key genes and functional modules.

Further information on the methods related to uncovering the control elements, interactions, and functional annotation of TCMIN is provided in Supplementary Text S2.

### General statistical analyses

General statistical analyses on observed differential gene expression used one-way ANOVA, Tukey’s HSD (Honestly Significant Difference) test, and applied Bonferroni correction using R version 2.15.1 (Supplemental Text S3). Significance testing of demographic and other potential confounding variables also used ANOVA and pairwise t-tests, as well as pairwise Chi-squared tests, and the effect size of intergroup differences was calculated with the Pearson product-moment correlation coefficient measured on a standard scale, where values of ±0.20, ±0.50, and ±0.80 can be considered small, medium, and large effect sizes, respectively (R version 2.15.1). These procedures properly account for differences in group size and any overlap in group membership.

## Results

### Identification and annotation of Transcriptomic (T) biclusters

The expression data of 34,602 transcribed genes in blood samples from 459 individuals with creative-reliable (*n* = 125), organized-reliable (*n* = 241), or emotionally unregulated (*n* = 93) personality profiles were compared for differential gene expression. We found little variability for most gene transcripts, as expected in this healthy population from the general population of Finns. Accordingly, the 1500 genes that were most variably expressed across personality profiles were selected for further analysis (Fig. 1). Bootstrap analysis of the subjects, where the expression of each of the 1500 genes were averaged for all individuals within the same network, indicated that the three personality groups differ in their gene expression (F = 198.97, *p* < 0.0001, ANOVA, Tukey HSD test *p* < 0.01 for all pairwise combinations after correction for multiple tests). The interactions of personality profiles with the transcriptomic gene expression were significant and strong (creative vs unregulated effect size *r* = 0.97; creative vs organized effect size r = 0.91; organized vs unregulated effect size *r* = 0.85), confirming the expected order of the effects of self-regulation on gene expression (creative > organized > unregulated) (Supplementary Text S3 and Table S1).

The differential gene expression related to personality was not attributable to covariates of the personality profiles (Supplemental Text S4). The interactions of personality with age and body mass index were non-significant and weak (effect sizes *r* < 0.0004) (Supplementary Table S2). The creative profile group had more women (*n* = 87) than men (*n* = 38), as is typical (Supplementary Text S4), so the interaction of personality groups with gender was significant (Chi-square, df = 2, *p* = 2.2E-04) (Supplementary Table S2).

**Fig. 2:**
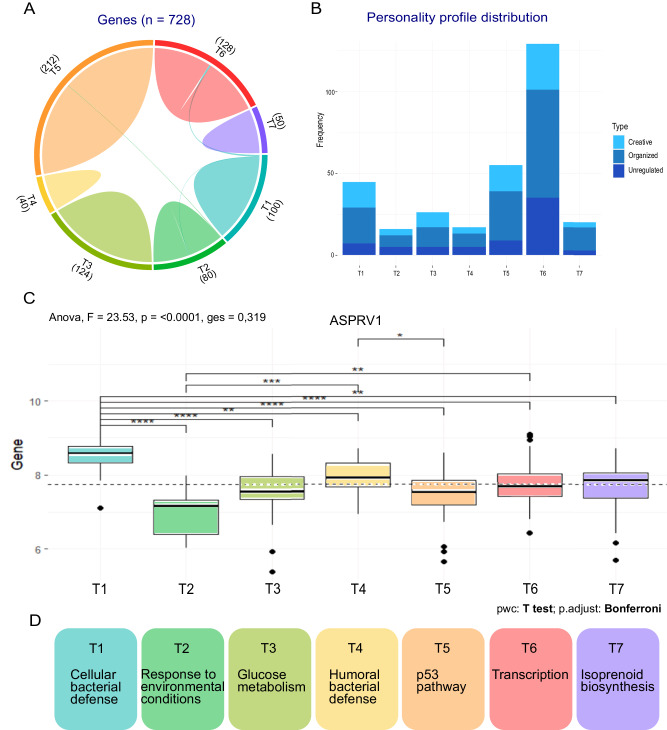
Analysis and functional annotation of transcriptomic biclusters. **A** Distribution and shared genes among T biclusters. **B** Distribution of the personality profiles (creative, organized, and unregulated as described in Zwir et al. 2021) across the T-biclusters. **C** Differences in ASPRV1 expression when comparing among T biclusters. **D** Functional annotation of the seven T-biclusters.

We identified seven transcriptomic (T)-biclusters (i.e., groups of genes in particular subsets of subjects) (T1-T7, Fig. 2A) among the 1500 variably expressed genes using PGMRA with a fixed number of 7 clusters (Supplementary Table S3). These biclusters recovered subjects occurring in more than one cluster (i.e., had more than one group of expressed genes), as permitted by PGMRA’s fuzzy clustering algorithm (Fig. 2A). The proportions of each personality profile differed little between the seven T-biclusters (Fig. 2B). The number of subjects in each T-bicluster varied, with T6 being the largest (*n* = 129) and T2 the smallest (*n* = 16) (Fig. 2C).

#### Variation in gene expression among subjects from different T-biclusters

The T-biclusters had little or no overlap in transcribed genes except for ZNF397, PIK3CB, JUN, PITPNA, SNX13, and LOC646527 (Fig. 2A). The number of genes in each T-bicluster varied, with T5 being the largest (212) and T7 the smallest (50) (Fig. 2A, Supplementary Table S3). We found significant differences in the average expression of each gene between T-biclusters, as illustrated for the gene ASPRV1 (Fig. 2C, Supplementary Tables S4, S5).

#### T-biclusters have distinct biological functions

The functional annotation of the genes in each T-bicluster revealed distinct, well-defined functions (Fig. 2D, Supplementary Table S6). These included cellular defense against bacteria (T1), reactivity to environmental conditions (T2), glucose metabolism (T3), humoral defense against bacteria (T4), regulation of p53 (T5), transcription (T6), isoprenoid biosynthesis, including cholesterol, acetyl-CoA, coenzyme Q10 (T7). Each cluster had diverse gene biotypes (e.g. protein-coding genes, ncRNAs, pseudogenes, antisense), with the only exception of T6, which contained 96% protein-coding genes.

### Co-expression of GE and T genes in shared brain regions (GETs)

**Fig. 3:**
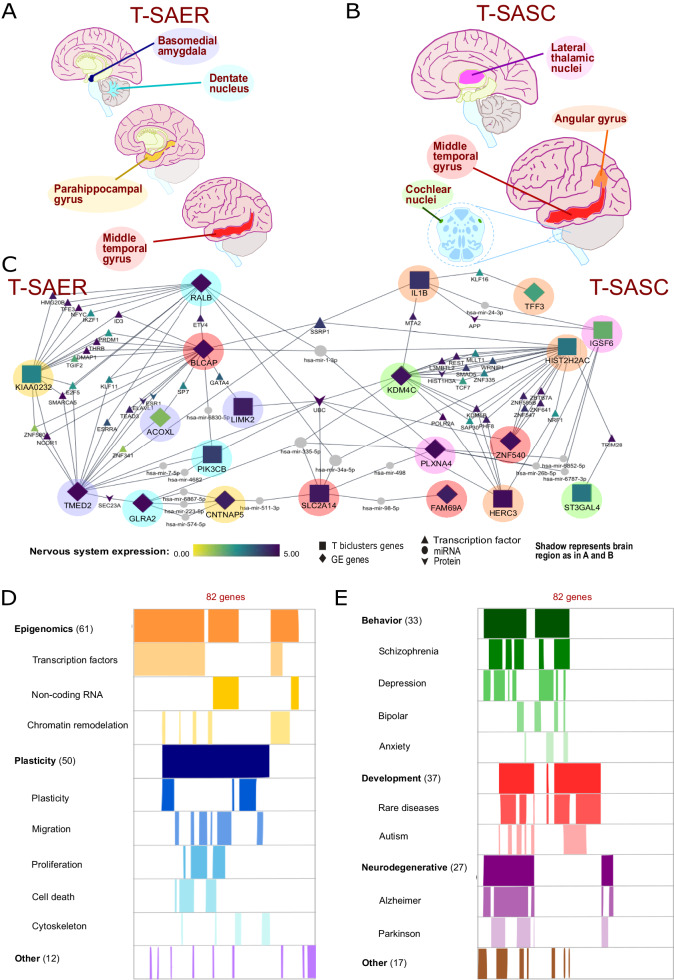
Analysis of T-SAER GET, T-SASC GET and T-SAER-SASC networks. **A** Brain regions linked to the genes from T-SAER GET. **B** Brain regions linked to the genes from T-SASC GET. **C** Integration of T-SAER and T-SASC minimum networks into an 82 genes network (called T-SAER-SASC) by the six shared genes in the central axis.* **D** Most relevant gene functions obtained from the existing literature, results could be summarized into epigenomic, plasticity and other functions. **E** Behavioral diseases, developmental diseases and neurodegenerative disorders related with the genes in the integrated T-SAER-SASC network. *Node shapes represent the type of gene as displayed in legend at bottom of the subfigure (**C**). The variation in color signifies the level of specificity of gene expression of each molecular type in the nervous system according to the values from the STRINGify database. The gradient of nervous system specificity varies from very high (purple), high (blue), intermediate (brown), low (green), to very low (yellow). Nodes in grey color lack information on tissue expression specificity in STRINGify. The circular shadings represent the different brain regions as colored in subfigures (**A**, **B**).

PGMRA biclustering methods identify genes in specific subjects, so we could test for relations of genes from the transcriptome and the genome in the same subjects. We found that the 728 variably transcribed genes in the 7 T-biclusters (Figs. 1C, 2B) did not overlap directly with any of the 972 genes in the personality-associated GE networks (Fig. 1D). To test for indirect interactions, we tested for possible co-localization in the same brain regions of the genes identified in both the transcriptomic and the genomic biclusters using PGL (Fig. 1E). The analysis of the brain regions in which these variably transcribed genes were significantly co-expressed (Fig. 3A–E, Supplementary Table S7) identified 9 different brain regions shared by GE and T genes and organized in four Genomic-Environmental-Transcriptomic (GET) subsets (Fig. 1F). Put another way, the genes in GETs shared one or more specific brain regions in which they were overexpressed (Fig. 3A–E, Supplementary Table S7).

Specifically, overexpressed transcripts were colocalized in the frontal cortex with genes from the genomic network for emotional reactivity (T-ER) (Supplementary Tables S7, S8). Other transcripts were colocalized in the corpus callosum with genes from the genomic network for self-control (T-SC) (Supplementary Tables S7, S8).

Genes associated with interactions between Self-awareness and Emotional Reactivity (T-SAER) were co-localized with transcripts overexpressed in four brain regions: the basomedial amygdala, cerebellar dentate nucleus, parahippocampal gyrus, and middle temporal gyrus (Fig. 3A, Supplementary Table S8). As expected, these brain regions were jointly involved in the self-regulation of anxiety (Supplementary Text S5 and Table S8).

Genes associated with the interactions between Self-awareness and Self-control (T-SASC) were co-localized with transcripts overexpressed in another four brain regions: the angular gyrus, middle temporal gyrus, lateral thalamic nuclei, and cochlear nucleus in the brainstem (Fig. 3B, Supplementary Table S8). As expected, these brain regions are jointly involved in self-awareness, particularly the production of meaningful concepts, cross-modal symbols, creative ideas, and figurative language (Supplementary Text S5 and Table S9).

### Network analysis of GET genes

To better understand the neurobiological relationships between the GE and the T genes, we performed a network analysis at three different levels of biological interaction in the GET networks: gene-miRNA interactions, gene-TF interactions, and gene-gene interactions for the T-SAER and T-SASC genes (Supplementary Text S6, Fig. S1, S2). This uncovered the hidden minimum molecular network that linked all genes within each GET (Fig. 3C, Supplementary Figs. S3, S4). We found that the interactions of GET genes create small regulatory networks that function as interactive modules because they link functional genomic variation, environmental responses, transcriptional and post-transcriptional processing in brain, as described below.

#### Genes for neuronal plasticity in the T-SAER network

The resulting T-SAER network was comprised of 45 genes (Fig. 3C, Supplementary Fig. S3, Table S9). These included 10 out of the 11 seed GET genes, 20 TFs, 10 miRNAs and 4 protein-coding genes (Supplementary Figure S3, Table S9). Functional annotation showed that 51% of these genes, including two zinc-finger proteins (ZNF580 and ZNF341), were involved in positive or negative modulation of cytoskeletal and/or synaptic plasticity (Supplementary Text S5 and Table S9).

The other 49% of the genes were mostly positive and negative regulators of epigenomic events (26%) and/or immune and stress responses consistent with their expected role in the interaction of self-awareness and emotional reactivity (Supplementary Table S9 and Text S6).

#### Genes for epigenomics and inflammatory response in the T-SASC network

The T-SASC network comprised 43 genes (Supplementary Fig. S4, Table S9). These included 13 seed GET genes, 19 TFs, 9 miRNAs and 3 protein coding genes (Fig. S3). In contrast to findings for the T-SAER network, in the T-SASC network we found that 44% of the 43 genes were involved in epigenetic events, including changes in the structure, function, and regulation of chromatin, and changes in gene expression through epigenesis (Supplementary Table S9 and Text S6).

21% of the 43 T-SASC genes were involved in processes related to plasticity (Supplementary Table S9). For example, TRIM28 controls the expression of transposable elements implicated in the regulation of human brain evolution and neurological disorder. The remaining 35% of the genes in the T-SASC network involved glucose transport.

#### Joint Control Hub of the GET networks

The T-SASC and T-SAER networks were found to share three miRNAs (hsa-miR-1-3p, hsa-miR-335-5p, and hsa-miR-34a-5p) and three protein-coding genes (SLC2A14, SSRP1, and UBC). These six genes allowed the integration of both GET interactive modules into a single information-processing network composed of 82 genes (T-SAER-SASC network, Fig. 3C).

#### Modal functions of the combined T-SASC-SAER network

Tissue-specific values of the genes in both GET networks showed a strong specificity for expression in the nervous system (Supplementary Figs. S3, S4, Table S10). Most of 82 genes in this network regulate neuronal plasticity (61%) and/or have epigenomic functions (74%) (Fig. 3D, Supplementary Table S9 and Text S7). Epigenomic functions include processes related to chromatin remodeling or activity of ncRNAs and TFs. Plasticity included neuronal and synaptic plasticity as well as processes related to cellular migration, proliferation, death, or the cytoskeleton (Supplementary Table S9).

#### Risk of neuropsychiatric disorder with T-SAER-SASC dysfunction

Expression of the constituent genes of the T-SAER-SASC network was enriched in the brain (Supplementary Table S10). Their mutation or dysfunction was associated with increased risk of neuropsychiatric, neurodevelopmental, and neurodegenerative disorders (Fig. 3E). 40% of the network’s 82 genes were associated with increased risk of behavioral disorders, including schizophrenia, depression, and less often bipolar or anxiety disorders. Developmental diseases were associated with 45% of 82 genes, including autism and various rare conditions (e.g., Fragile X syndrome, Williams syndrome, Angelman syndrome). Neurodegenerative disorders, such as Alzheimer’s and Parkinson’s disease, were associated with 33% of 82 genes (see Supplementary Text S5, Table S9).

### A broader perspective on temperament-character integration

To uncover the broader underlying regulatory network that we had hypothesized, we examined all interactions at both miRNA-gene and gene-gene levels for the six genes in the control hub of the integrated T-SAER-SASC network. Using bioinformatic resources, we found that the six coordinating genes directly interact with 4190 genes, including 3919 protein-coding genes, 198 miRNAs, 38 pseudogenes, and 35 other ncRNAs (Supplementary Tables S11, S12).

**Fig. 4:**
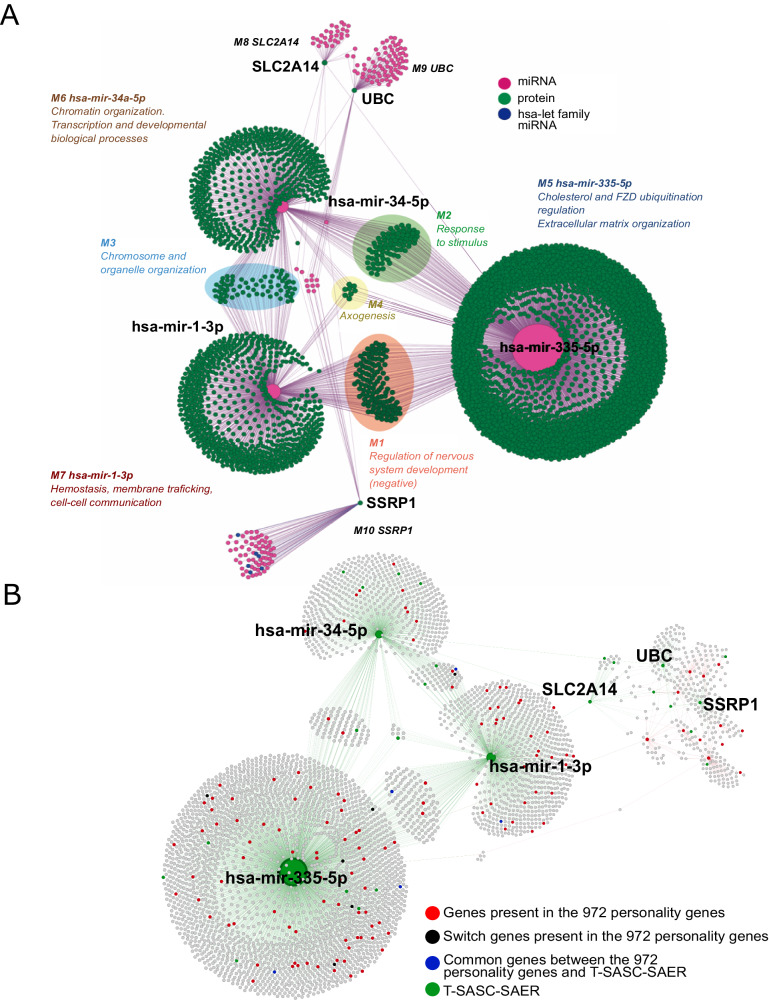
Temperament-character molecular integration regulatory gene network (TCMIN). **A** PPI and miRNA-gene interaction network. * **B** Extension of the TCMIN with personality lncRNAs from Zwir et al. 2021.# * Modularity analysis and node importance were calculated using community detector algorithm and eigenvector centrality with Gephi. Node size depends on the node eigenvector centrality. Node color represents the molecule type, pink for microRNAs, green for proteins, blue for hsa-let-miRNA family (Supplemental Text S8). Ten different functional modules are indicated by a different color legend. Modules regulated by two or three hub-miRNAs (hsa-mir-1-3p, hsa-mir-335-5p, and hsa-mir-34a-5p) are marked by an additional background color indicating shared regulatory functions: M1 (light orange), neural system development; M2 (light green), response to stimuli; M3 (light blue), nucleosome organization and epigenesis; M4 (light yellow), axogenesis. # Colored nodes represent the intersection fraction of the 972 (Zwir et al.) personality genes with the TCMIN. Different colors stand for different origin/functionality of the intersected genes: black indicates a personality switch gene, blue indicates communalities between the 972 personality genes and the T-SASC-SAER network; red indicates common genes between TCMIN and the 972 personality genes that are not switch genes or genes from the T-SASC-SAER network. In green appear T-SASC-SAER genes that are not part of the 972 personality genes.

These 4190 genes are organized in 10 functional modules (Fig. 4A, Supplementary Text S7). Interactions among these modules gives rise to the temperament-character molecular integration network (TCMIN) (Fig. 4A, Supplementary Tables S11, S12).

To evaluate the role of interactions of the miRNAs in TCMIN more fully, their known interactions with personality-related ncRNAs were added (Supplementary Table S13), extending the number of genes in TCMIN to 4376 in addition to the 6 genes in the coordinating hub (Fig. 4B, Supplementary Table S14). These genes in the extended TCMIN included 3919 protein-coding genes, 371 miRNAs, 38 pseudogenes, and 48 other ncRNAs. Of the 972 personality-associated genes known from our prior GWAS, we had already identified 129 personality-associated genes coordinated by the smaller TCMIN (Supplementary Table S11) and identified another 14 personality-associated lncRNAs coordinated in the extended TCMIN (Supplementary Table S15). To describe the functional organization of the molecular integration network (Supplementary Text S8), these modules are described systematically as follows.

#### MiRNAs are key control elements of the TCMIN network

Network analysis revealed that hsa-miR-1-3p, hsa-miR-335-5p, and hsa-miR-34a-5p are the key regulators of the TCMIN (Fig. 4, Supplementary Text S8, Table S12, S16). Each of these miRNAs interacted with hundreds of protein-coding genes either individually, in pairs, or all three together (Fig. 4A, Supplementary Text S8).

The 2413 genes in the module of genes that interacted only with miRNA *hsa-miR-335-5p* (Module 5 in Fig. 4A) are involved in the regulation and maintenance of cellular structure and function, including the control of lipid levels (e.g., cholesterol), the modification of key signaling receptors (e.g., FZD ubiquitination), and extracellular matrix organization (Supplementary Tables S11, S17).

The module of 577 genes interacting only with miRNA *hsa-miR-34a-5p* is mostly involved in regulation and maintenance of gene expression during development, including the control of chromatin structure, transcriptional activation or repression, and the orchestration of complex developmental processes (Module 6 in Fig. 4A, Supplementary Tables S11, S17).

The module of 708 genes that interacts only with miRNA *hsa-miR-1-3p* regulates and maintains cellular and physiological processes, including hemostasis, membrane trafficking, and cell-cell communication (Module 7 in Fig. 4A, Supplementary Tables S11, S17).

#### The three hub miRNAs act in coordinated manner over specific sets of genes

The 3 hub miRNAs also control smaller, more specific groups of genes in pairs or altogether. Module 1 of the TCMIN network (Fig. 4A) is comprised of 122 protein-coding genes modulated jointly by *hsa-miR-1-3p* and *hsa-miR-335-5p*. Module 1 is enriched with genes involved in nervous system development, especially axogenesis and neurogenesis (Supplementary Table S17). Variants in these genes are associated with neuropsychiatric disorders (Supplementary Text S8 and Table S18).

Module 2 of TCMIN is comprised of 80 protein-coding genes modulated jointly by miRNAs *hsa-miR-34-5p* and *hsa-miR-335-5p*. It was enriched for genes coding for sensory responses to diverse types of stimuli, including mechanical stimuli, gravity, oxidative stress, hormones, or various chemical substances (Supplementary Table S17). Variants of all genes in this group have been linked to phenotypes with modifications of stimulus reactivity and neuronal activity in brain (Supplementary Table S18). For example, SYT1 is a stimulus-dependent gene that regulates both the rate and the size of synaptic vesicles formed during endocytosis; it is also required during exocytosis.

Module 3 is comprised of 67 protein-coding genes modulated jointly by miRNAs *hsa-miR-1-3p* and *hsa-miR-34-5*. The M3 group was enriched only in GO terms related to cellular component organization regulation (Supplementary Table S17). All genes in this group were involved neuronal development and psychiatric anomalies or chromatin and organelle organization, such as SMARCC1, which stimulates the remodeling activity of nucleosomes (Supplementary Table S18).

Module 4 of TCMIN was the only module regulated by all three miRNAs of the central hub. It was comprised of 8 protein-coding genes that were enriched in axogenesis (Supplementary Table S17). Variants in these genes are documented to alter axonal growth, fasciculation, and synaptogenesis (Supplementary Table S18). For example, L1CAM plays a crucial role in the formation of major axonal tracts such as the corticospinal tract and corpus callosum (Supplementary Table S18 and Text S8).

#### Protein-coding genes SLC2A14, UBC and SSRP1 interact with small clusters of miRNAs

The three protein-coding genes in the molecular integration hub interact with miRNAs in Modules 8, 9, and 10. Especially interesting is Module 10 (M10-SSRP1) which consists of 69 miRNAs that interact with SSRP1, which influences neuronal differentiation, development, synaptogenesis, neuronal excitability, epigenesis, and risk of psychopathology (Supplementary Text S8).

#### Presence of many plasticity-related genes in TCMIN

Our manual curation process revealed many zinc-finger genes (*n* = 143) in the TCMIN (Supplementary Fig. S5, Table S12), which are related to the development, maintenance, and functioning of neuronal cells. Zinc-fingers have been found to play a vital role in neural development, synaptic plasticity, learning and memory, and in the regulation of neurotransmitter receptors, ion channels, and other signaling molecules that are essential for proper brain function (Supplementary Text S8).

Moreover, histones (*n* = 61) and ubiquitins (*n* = 40) (Supplementary Table S12) were exclusively present in the M6 (hsa-miR-34-5p only) and the M3 (hsa-miR-34-5p together with hsa-miR-1-3p) clusters (Fig. 4A). These clusters are involved in chromatin organization (M6) or chromosome and organelle organization (M3) (Supplementary Fig. S5). Manual curation also identified 49 genes associated with neuronal system development, 25 genes related to synapses, and 38 pseudogenes (Supplementary Table S12).

#### TCMIN genes act in a dynamic and coordinated way

TCMIN genes show a noteworthy enrichment in genes related to liquid-liquid phase separation (LLPS). This process enables specific RNAs and proteins to create membrane-less organelles. As hypothesized, this enrichment is supported by chi-square tests for no enrichment with *p*-values of 1.45E-09 for reviewed LLPS-RNAs and nearly 0 for high-throughput LLPS-RNAs (Supplementary Tables S14, S19–21; Text S2). Genes in TCMIN included 32% of the 72 previously known LLPS-RNAs in the comprehensive RPS database, which is greater than in the entire human genome (0.12%, Supplementary Tables S14). The enrichment is even greater when based on the high-throughput RPS database of 9571 LLPS-RNAs: The TCMIN contains 1699 high-throughput LLPS-RNA genes, so 39% of the genes in TCMIN are related to LLPS compared to 15.7% in the entire human genome (Supplementary Table S14).

The genes directly regulated by the three miRNAs in the hub of TCMIN include many related to LLPS. 51% of the genes regulated by miRNA has-miR-1-3p are high-throughput LLPS-related RNAs, as are 37% to 39% of the genes regulated by the other two hub miRNAs. More than half of the genes in functional modules regulated by multiple hub miRNAs are high-throughput LLPS-related RNAs: M1 for regulation of nervous system development with 59.0%, M2 for stimulus response with 57.5%, M3 for chromosome and organelle organization with 52.9%, and M4 for axogenesis with 62.5% (Fig. 4, Supplementary Text S8 and Tables S19, S20). These LLPS-related RNAs have been shown to regulate stress responses, homeostasis, development, and risk of disease (Supplementary Text S8 and Table S14).

### Conservation of the six hub genes that regulate TCMIN

The TCMIN analysis demonstrated that the 3 miRNAs were key control elements in the integration network (Fig. 4A, Supplementary Table S12), regulating specific sets of genes individually and collaboratively. To investigate the evolutionary conservation of these key miRNAs, we extracted orthologs from 151 genomes across the tree of life (Supplementary Fig. S6). Our results revealed that hsa-miR-34a-5p was the most conserved miRNA, with orthologs present in 96 different genomes, 89 of which were 1-to-1 orthologs (Supplementary Table S22). The other two were also well-conserved: hsa-miR-1-3p was present in 93 different genomes, with 84 1-to-1 orthologs (Supplementary Table S22), and hsa-miR-335-5p with 85 orthologs, 82 of which were 1-to-1 orthologs in different genomes (Supplementary Table S22). The least conserved, hsa-miR-335-5p, was also found in many mammals, ranging from old-world monkeys to alpaca or rabbits. Among all three miRNAs, the most conserved, hsa-miR-1-3p, was also present in the widest spectrum of the tree of life including one reptile, some birds, and marsupials.

All three miRNAs were only found together in 28 mammalian genomes (Supplementary Table S23). There was variation in the percentage of sequence similarity between the query and target in all cases, except for identical target sequences in three hominid genomes (chimpanzees, bonobos, and gorillas). Of these 28 genomes, eight were primates, including three of the hominid suborders *(Pan troglodytes, Pan paniscus*, and *Gorilla gorilla gorilla*), three of the suborder Similiformes (*Chlorocebus sabaeus, Macaca fascicularis*, and *Macaca mulatta*), and one of suborder Strepsirhini (*Microcebus murinus*). Only one Feliforme genome (*Panthera pardus*) appeared, along with six Artiodactyla genomes, eight Rodentia genomes from different suborders, two Carnivora genomes from different Canidae suborders, two Perissodactyla genomes, and one Proboscidea genome from the Elephantidae suborder.

The 3 protein-coding genes in the control hub are also highly conserved. Further information is provided in Supplementary Text S9.

### Relation of TCMIN genes with previously described personality genes

When we compared the 4190 genes from the TCMIN with personality-associated genes, we found that the three hub-miRNAs from the TCMIN coordinate 129 genes from the 972 personality genes (Supplementary Table S11). It is noteworthy that among these 129 genes, we identified six “switch genes” (BMP7, NR3C2, RGS13, VPS8, ZNF503, and SLC44A5), which are associated with differences in health status among individuals with similar character profiles (Fig. 4B, Supplementary Text S10). Except for BMP7, all other 5 switch genes are in the cluster regulated by hsa-miR-335-5p (Module 5 as shown in Fig. 4B and Supplementary Table S12).

Because TCMIN was constructed solely based on gene-miRNA, TF-gene, and protein-protein interactions, we further investigated the relation of TCMIN with personality-related lncRNA genes. Personality-related lncRNAs were of special interest because some of them are unique to modern humans [7], so we searched for their interactions with TCMIN genes. We found 102 interactions involving 20 personality-associated lncRNAs, seven of which are unique to humans (CASC15, LINC00472, LINC01450, LMCD1-AS1, UGDH-AS1, ZNF503-AS1, and ZNF571-AS1) (Supplementary Fig. S5, Table S13). The link between these 20 personality-associated lncRNAs and the TCMIN was facilitated by small gene clusters coordinated by SLC2A14, UBC, SSRP1, and 61 additional miRNAs (Supplementary Fig. S12, Text S10). The names and biotypes of the 4376 constituents of the extended TCMIN network are described in Supplementary Table S15.

## Discussion

### Significance of our findings

Our integrative analysis of the genomics, transcriptomics, and proteomics of human personality has allowed us to shed new light on three long-standing scientific mysteries: (i) the relations between mind and body [112–116], which we find to be interactive aspects of a undivided whole, (ii) the role of self-awareness of a person’s participation in the unity of all existence [117–120], which we found to regulate gene expression and epigenesis to mediate all aspects of well-being (physical, mental, social, and spiritual), and iii) the growing evidence that ribonucleic acids were the progenitors of all forms of cellular life on Earth through their capacities for liquid-liquid phase separation (LLPS) and catalysis of protein synthesis [85, 86, 121, 122]. LLPS-related RNAs create membrane-less compartments in which they can maintain the conditions needed for self-replication by synthesizing their own constituents, which we found is highly conserved in evolution from single cells to self-aware humans. We will first briefly summarize these highlights and then discuss them.

Regarding the nature of mind-body interactions and their role on regulating gene expression, this is the first study to uncover the complex multi-modular organization of gene regulatory networks associated with human personality. There have been no prior combined genome-wide and transcriptome-wide studies of human personality to our knowledge. The one published study of gene expression related to personality in humans focused on a priori-defined sets of genes involved in inflammation in a convenience sample of 117 students with no information on the genotypic-phenotypic structure of the personality measures used [123]. A few small studies in non-human primates focused only on anxious temperaments and a priori-defined sets of brain regions and genes, which by design cannot characterize the organization of the complex regulatory networks underlying human personality [124–126].

We found that personality is associated with a molecular integration network that regulates multiple specialized functional modules that turn one another on and off via reciprocal interactions. We found that transcribed RNAs actively inform complex multi-modular networks that regulate all aspects of living systems, as proposed by holographic models of mind and body as interactive components of one whole that cannot be reduced to separate objective and subjective parts [117–119].

Holographic models are three-dimensional, not hierarchical, as expected from our prior finding of three systems of learning and memory underlying human personality [64]. Associative conditioning of basic emotions (temperament) is the preconscious body component of personality, whereas the conscious mind (character) has two qualitatively different components that Chalmers and others have distinguished as Mind 1 (i.e., intentional self-control, the analytical mind that reasons hierarchically and deterministically from assumptions and observations) and Mind 2 (i.e., creative self-awareness, which is intuitive, holographic, and open-ended) [80, 115, 127, 128]. Our findings support the quantum physicist David Bohm’s proposal that RNAs transfer active information that guides adaptive cellular processes holographically [117–119].

We also found that LLPS-related RNAS were greatly enriched in this molecular integration network: they accounted for 39% of more than 4000 genes in the network compared to 16% of the genes in the total human genome. They comprised most of the genes in functional modules for regulation of brain functioning, adding new evidence about their role in the evolution of increasing plasticity, consciousness, and creativity over the past 4 billion years. Consequently, human personality and its self-regulatory networks for gene expression are not only dynamically adaptive and self-organized, but also health-promoting, collaborative, open-ended, and creative, as we hypothesized and described in the introduction based on prior theory [4, 6, 16, 26, 69] and empirical research [7, 8, 64, 65, 67, 68].

Put another way, human functioning depends on personality configurations that indicate the level of self-awareness (i.e., insight) of a person’s perspective on the fundamental unity of existence and what is healthy, satisfying, and meaningful. In turn, a person’s self-awareness shapes and guides the coherence of the interactions among three major systems of learning and memory underlying human personality. Hence our findings about the regulation of gene expression confirm that the healthy functioning of human personality is an open-ended integrative biopsychosocial process [67–69, 71], which cannot be reduced to simpler explanatory models that are deterministic [6, 16].

Specifically, we compared the regulation of gene expression in people with three levels of self-aware consciousness: (i) “*unregulated*”: people with little or no self-awareness (i.e., emotionally reactive and motivated primarily their habits, traditions, and irrational desires, (ii) “*organized*”: egocentric and materialistic individuals who learn by using intentional self-control to regulate their habits and to set self-serving goals using their analytical intellect, and (iii) “*creative*”: allocentric and self-transcendent individuals who use self-awareness to perceive how to live in meaningful harmony with others, nature, or the universe as an inseparable aspect of a greater whole. We confirmed our prediction that regulation of gene expression was strongly influenced by self-awareness (creative > organized > unregulated), just as we had previously found that self-awareness was strongly associated with all aspects of well-being (physical, mental, social, and spiritual) [7, 64, 67, 72, 129]. Our findings from transcriptomics show that deterministic models [113, 114] that correspond to the reductionistic perspectives of people with unregulated or organized personalities are inadequate for understanding the fundamental properties of living systems, such as being self-replicating, self-organizing, and open-ended [6, 7, 16, 65, 66, 115, 130, 131].

### The regulation of gene expression networks by human temperament and character

Using network analysis of combined genomic, transcriptomic, and proteomic information, we discovered that human personality orchestrates interactions between two multi-modular systems regulating gene expression: an extrinsic (bottom-up) system and an intrinsic (top-down) system. The extrinsic gene expression system regulates plasticity of rapid responses to salient sensory and emotional signals about environmental conditions in a way that is shaped by a person’s temperament. In contrast, the intrinsic system regulates conceptual and figurative interpretation of the meaning of experiences in a narrative context that is shaped by the identity and character of the person.

The integration of the extrinsic and intrinsic systems by reciprocal feedback between the salience and meaning of changing internal and external conditions shapes and coordinates gene expression for several specialized adaptive functions that are encrypted in the genome but only expressed in the transcriptome in response to changing conditions in a biopsychosocial context. Thus, the level of a person’s self-awareness (SA) is a crucial influence on self-regulation of both emotional reactivity (ER) in the extrinsic system and self-control (SC) in the intrinsic system. These findings suggest the hypothesis that self-regulated differences in gene expression strongly mediate the relationship between an individual’s personality and their health, which can be tested in future studies of a wide range of physical, mental, and social health outcomes.

### Discovery of the Genomic-Environmental-Transcriptomic regulatory networks

In our network analysis we uncovered the extrinsic network by identifying variably transcribed (T) genes that were expressed in the same regions with genes associated with interactions between the genomic networks for self-awareness (SA) and emotional reactivity (ER). We identified this network initially as the Transcriptomic-Self-Awareness-Emotional Reactivity (T-SAER) network. Functional annotation confirmed that brain regions specified by the T-SAER network were involved in self-regulation of anxiety by genes that regulate neuronal plasticity (Fig. 3A).

Likewise, we uncovered the intrinsic network by identifying variably transcribed genes colocalized in brain regions with genes associated with interactions between the genomic networks for self-awareness and self-control. We identified it initially as the T-SASC network (Fig. 3B). Functional annotation revealed that the brain regions specified by the T-SASC network were co-expressed in brain regions for production of conceptual interpretation and figurative language in self-awareness. The genes in that network regulate epigenetic change and context-dependent adaptations. For example, they promote meta-stable adaptations, such as cooperation and creativity in states of well-being associated with an outlook of unity, or defensiveness and inflammatory reactions in states of ill-being associated with an outlook of separation [8, 61, 69].

Both the extrinsic and the intrinsic regulatory networks for gene expression are preferentially expressed in the nervous system. Likewise, in prior work we had found that temperament and character account strongly for individual differences in fMRI connectivity networks involving the prefrontal cortex and its connections with other brain regions in humans [8]. The identified connections included the association of temperament with bottom-up networks for sensory and emotional reactivity (salience and ventral attention networks), and character with top-down networks for self-control (cingulo-opercular, fronto-parietal, dorsal attention networks) and self-awareness (default mode network) [8].

Our current findings about the role of personality in the regulation of gene expression correspond well to prior work on strong relations between personality as a psychosocial phenotype [61, 62] and its underlying systems of learning and memory [64, 65] and brain functional connectivity [8]. Thus, the organization of the gene regulatory systems associated with personality corresponds well to the organization of the dynamics of personality as an observable biopsychosocial phenotype associated with functional connectivity networks in the human brain.

### Discovery of the temperament-character molecular integration network

Our most important and novel discovery is the mechanism by which temperament and character orchestrates the reciprocal interactions between the extrinsic and intrinsic regulatory networks for gene expression that promote health and well-being. We found that these two networks shared six genes: 3 miRNAs (hsa-miR-1-3p, hsa-miR-335-5p, and hsa-miR-34a-5p) and 3 protein-coding genes (SLC2A14, UBC, and SSRP1). These six shared genes allowed the integration of both networks into a single information-processing network composed of 82 genes, which we designated as the T-SAER-SASC network (Fig. 3C). Functional annotation indicated that these genes operate as the core regulatory hub by which personality orchestrates neuronal plasticity, development, and epigenetic change to coordinate adaptive changes in gene expression and epigenesis. Information transfer from human self-awareness coordinates positive and negative regulators of functionally specialized modules. These reciprocally interactive modules turn one another on and off dependent on awareness of changes in the meaning and salience of internal and external conditions. The coordination of this activity choreographs and orchestrates a person’s unique pattern of adaptive functioning as an objective expression of their personality.

Specifically, we found that the three hub-miRNAs from the TCMIN (hsa-miR-1-3p, hsa-miR-335-5p, and hsa-miR-34a-5p) coordinate 129 genes interacting with the personality-associated genes identified by GWAS. It is noteworthy that we identified six “switch genes” (BMP7, NR3C2, RGS13, VPS8, ZNF503, and SLC44 A5) among them (Fig. 4B, Supplementary Table S12). These switch genes were previously described as genes related to changes in health status among individuals with similar character profiles [61]. Five of the six switch genes were in a specialized module regulated by miRNA has-miR-335-5p.

Likewise, the three protein-coding genes interacted with 3 clusters of another 61 miRNAs, each with distinct specialized functions. Altogether we found that these six genes act as a coordinating hub that interacts directly with 4190 genes organized in 10 specialized functional modules (Fig. 4A) that regulate the many processes underlying neuronal plasticity, development, and epigenetic change. The number of genes in TCMIN increased to 4376 when known miRNA interactions with lncRNAs were added, which is important for considering the emergence of self-awareness and creativity in the hominid lineage (Fig. 4B, Supplementary Table S14).

The presence of a small regulatory core provides an efficient way to integrate reciprocal feedback interactions within multi-modular gene expression networks that have widespread effects throughout the central nervous system and, in turn, on the person who is embedded in an even greater and dynamic context. Specifically, the presence of a central hub associated topologically with personality-related genes enables efficient information transfer from the self-awareness of an individual to the coordinating hub; this enables a self-aware person to shape and adapt to changing conditions in accord with their unique identity as expressed by their personality profile and related emotions, goals, and values [7, 69, 80].

The regulation of reciprocal interactions between the salience and meaning of changing events provides capacities for dynamic self-organization that is rapid and reversible, as well as for epigenesis that involves enduring changes in biopsychosocial development [69, 79, 80]. This corresponds precisely with the defining characteristics of personality as the dynamic organization within the person of the biopsychosocial systems that shape and adapt their responses to ever-changing internal and external conditions [79, 80].

It is important to recognize that our finding that the level of a person’s self-awareness accounts for their capacity to regulate gene expression does not mean that these regulatory processes are simply intentional choices, that would depend on slow deliberation while awake. Rather the character configurations predictive of differences in regulation of gene expression involves shifts in a person’s intuitive perspective, which is strongly associated with differences in brain functional connectivity at rest and is highly stable whether a person is awake, asleep, or anesthetized [8]. For example, people with the creative character profile have a spontaneous perspective based on an outlook of unity, which motivates them automatically to be self-directed, cooperative, and self-transcendent, including the ability to let go of intentional struggles. In full form, such an advanced state of consciousness has been described as “automatic intelligence”, “choiceless awareness”, or “awareness of unity” [66, 69, 132]. Consequently, all aspects of health can be promoted by growth in self-awareness [69].

To understand how these regulatory processes operate automatically while guided by meta-stable insight from self-aware cognitive processes unique to humans, we will discuss the structure and function of the molecular integration network of temperament and character more fully. Specifically, we will next discuss its collaborative components and their functional roles from an evolutionary perspective and then from the perspective of functional neuroanatomy.

### The evolution of increasing complexity, plasticity, and consciousness

Phenotypic evolution is linked to the evolution of the regulatory systems for gene expression [27]. Therefore, we made predictions about the importance of specific types of genes likely to be conserved throughout the incremental steps in the evolution of the regulatory mechanisms of gene expression, including four major evolutionary steps: (1) liquid-liquid phase separation (LLPS), which is a prebiotic molecular process by which protein-coding and non-coding RNAs form membrane-less compartments and which has been conserved in intracellular processes of all forms of cellular life [85–90]; (2) chromatin organization and TF binding to DNA, which is associated with the development of multicellular organisms and conserved for its ability to interpret information encrypted in DNA in a flexible, context-dependent manner [27, 28, 91]; (3) miRNA-gene interactions, which are associated with the marked increase in modularity and complexity in animals; and (4) miRNA-lncRNA interactions, which coordinate the expression and co-localization of genes in distributed regional connectivity networks associated with self-aware consciousness and creativity in modern humans [7, 65].

We tested and confirmed our predictions by finding evidence of the enrichment of each of the four predicted types of genes and their gene-gene interactions.

#### RNAs related to the process of *liquid-liquid phase separation* (LLPS)

LLPS-related RNAs organize specialized functions in membrane-less organelles in the cytoplasm or nucleoplasm of all cells. They compartmentalize and coordinate biochemical reactions, regulate gene expression, and participate in signaling.

We confirmed that LLPS-related RNAs were significantly enriched in the molecular integration network using RPS, a comprehensive database of RNAs involved in LLPS [88]. LLPS-related genes accounted for 39% of the 4376 genes in TCMIN compared to 15.7% of the 61,035 genes in the total human genome (Supplementary Table S14). 51% of the genes regulated by miRNA hsa-miR-1-3p are high-throughput LLPS-related RNAs, as are 37% to 39% of the genes regulated by the other two hub miRNAs. More than half of the genes in functional modules regulated by multiple hub miRNAs are LLPS-related RNAs, including modules for regulation of nervous system development (59%), stimulus reactivity (58%), chromosome and organelle organization (53%), and axogenesis (63%) (Fig. 4).

A key advantage of LLPS-based regulation is that it enables rapid, precise, and reversible assembly and disassembly of membrane-less organelles in response to changing conditions. They have been shown to be essential for regulating stress responses [133], maintaining homeostasis [134], and facilitating development [135]. Dysregulation of LLPS is closely associated with several diseases [136, 137].

#### TF – gene interactions

Sequence-specific binding of TFs to DNA is a key regulator of transcription and the formation of protein-protein interaction networks. We confirmed a prominent role for transcriptional regulation of gene expression within the molecular integration network. The majority (52%) of the 4376 genes in TCMIN encoded proteins involved in a variety of protein-gene and protein-protein interactions that were associated with known personality-related genes. The other non-coding gene variants were regulatory RNAs (1699 LLPS-RNAs, 190 miRNAs, 186 lncRNAs), and 38 pseudogenes.

The protein-coding genes were organized in specialized functional modules organized by miRNAs (see Fig. 4A). These include modules for regulating the posttranslational modification of key signaling receptors (module 5, hsa-miR-335-5p with 2413 genes) and control of chromatin organization, transcription activation and repression, and embryogenesis (module 6, hsa-miR-34a-50 with 577genes). Cell-cell communication, as is needed in multicellular animals, is regulated by another 708 genes interacting only with miRNA hsa-miR-1-3p in another specialized module of TCMIN (module 7, Fig. 4A, Supplementary Tables S12, S17).

#### MiRNA-Gene interactions

In the post-transcriptional phases of gene expression, miRNAs are widely considered to have the key regulatory role [27, 28, 44]. Specifically, miRNAs are hypothesized to be key for the evolution of regulatory processes associated with increasing neuronal complexity and consciousness in bilaterians (i.e., animals with bilateral symmetry as an embryo), particularly in the lineage of primates and hominids, which have exceptionally high numbers and diversity of miRNAs unique to them [27, 44, 46, 94]. We confirmed that miRNAs orchestrated the molecular integration network by coordinating interactions among more than 4000 genes in TCMIN, including more than 100 genes that directly overlap with known genes for interactions between self-awareness and other components of human personality (Fig. 4, Supplementary Table S12). The key regulators are the three miRNAs in the TCMIN that regulate functional groups of protein-coding genes individually, pairwise, or altogether. Our results suggest that these three miRNAs (hsa-miR-1-3p, hsa-miR-335-5p, and has-miR-34a-5p) play a crucial role in the regulation of the complex brain development underlying brain connectivity and human personality, as well as neuronal plasticity, allostasis, and epigenesis, and adult neurogenesis.

It is noteworthy that all three miRNAs in the human molecular integration network are only present with 100% similarity in three hominid species (chimpanzees, bonobos, and gorillas) and other vertebrate species vary in conservation corresponding to their levels of complexity, plasticity, and consciousness (Supplementary Fig. S6, Table S23). Orthologs were found only in some mammals and birds, 1 reptile, but not amphibians or fish (Supplementary Fig. S6).

#### (d) Interactions of personality-related ncRNAs with plasticity genes

Interactions among lncRNAs and miRNAs have been found to coordinate the expression and co-localization of genes in the human brain’s regionally distributed connectivity networks, which function efficiently when a person has well-developed self-awareness [7, 65, 138]. We confirmed that 20 personality-related lncRNAs interacted with 102 genes in TCMIN, including 7 personality-related lncRNAs unique to modern humans (Supplementary Table S13). The interactions between genes for personality and for neuronal plasticity were coordinated by the three protein-coding genes in the TCMIN control hub that organized small clusters of ncRNAs (miRNAs, lncRNAs, and antisense RNAs) (Supplementary Fig. S5). The interactions of personality-related ncRNAs involved special classes of plasticity-related genes in the TCMIN that have evolved in number and diversity in hominids [139], such as zinc-fingers, histones, ubiquitins, and genes related to neuronal system development and synaptic plasticity.

Overall, these findings showed that the molecular integrative network coordinates regulatory elements in all phases of gene regulation from transcription to translation and post-translational modification. These steps in the regulation of gene expression in humans reflects the incremental increase in the plasticity, complexity, and consciousness of organisms in the evolutionary lineage that can be traced from unicellular organisms to self-aware humans [66]. The symbiotic properties of adaptive processes in biological systems promote a synergistic triad of increasing plasticity, expanding consciousness, and adaptive functioning as a cycle of emergent creativity and virtuosity in the evolution of life forms [26, 69, 81].

However, to fully appreciate the complexity of the components of the molecular integration network associated with human personality, it is essential to retrace its network structure to appreciate how a core hub of six genes can coordinate many specialized modules in a human way. Human personality is a self-organizing expression of an individual’s personal identity that facilitates incremental growth of enduring goals and values as a person seeks to cultivate health, satisfaction, and meaning in their life. We need to ask what it is about the complex systems for learning and regulation of gene expression that account for the unique characteristics of human personality with self-awareness and open-ended creativity. For that we need to take functional neuroanatomy into account in interpreting the findings of our network analyses.

### Functional neuroanatomy of indirect but coherent genomic-transcriptomic relations

It is important to consider the neuroanatomy and neuropsychological functions of the brain regions that allowed us to identify the integrative molecular processes underlying the high level of plasticity, complexity, and self-aware consciousness observed in human personality and brain functional connectivity. As shown in prior work, the connectivity networks of human prefrontal cortex and its connections with parietal, temporal, limbic, and cerebellar hubs are crucial for a person’s rational self-governance and personality [8, 140–142]. The intrinsic (top-down) prefrontal networks for rational self-governance are strongly related to human character profiles, whereas extrinsic (bottom-up) prefrontal networks are strongly related to human temperament and emotional reactivity [8]. Our current findings about the brain regions at the foundation of the extrinsic and intrinsic networks of molecular integration help to clarify how the genetic networks regulate information processing and orchestrate the dynamic interactions between temperament and character in adapting to changing internal and external conditions.

The extrinsic network for interactions of self-awareness and emotional reactivity with regulation of variably transcribed genes was based on genes co-localized in four specific brain regions: the basomedial amygdala, cerebellar dentate nucleus, parahippocampal gyrus, and middle temporal gyrus (Fig. 3A). We confirmed that these brain regions are jointly involved in the regulation of anxiety [143–146] (Supplementary Tables S9). The connections and functions of the basomedial amygdala and hippocampal-middle temporal regions interact to translate emotions, especially fear and anxiety, into outcomes adaptive to the person’s self-awareness of their biopsychosocial context. The amygdala has the unusual role of screening and censoring its own input prior to parahippocampal processing [146, 147], so prior experiences that influence the state of the amygdala can bias perception by effects on what is salient because of selective context-dependent attention and unrealistic extremes of optimistic risk-taking or anticipatory worry and pessimism. For example, when a person is high in Novelty Seeking and enters a restaurant hungry, they notice only the food on people’s plates, but when they exit with hunger satisfied, only then are they likely to notice the faces of people at other tables [148]. When a person is highly harm-avoidant and already agitated, their startle reflexes are amplified, whereas when a person is low in Harm Avoidance and relaxed, their startle reflexes are reduced [149, 150].

In contrast, the intrinsic network for the interactions of self-awareness and self-control involved genes expressed in a different set of four specific brain regions: the angular and middle temporal gyri, the lateral thalamic nuclei, and the cochlear nucleus in the brainstem (Fig. 3B). We confirmed that all four regions in the intrinsic network are responsible for multimodal sensory processing, integration and interpretation of conceptual meaning, and dynamic mental representation for cross-modal symbols underlying metaphorical expression, creative ideation, narrative figural art, figurative language, and reading in self-aware consciousness [7, 151, 152]. The angular gyrus sits at the brain’s cross-roads for transfer of multimodal sensory information, including somatosensory, auditory, and visual inputs, as well as information on taste and smell. It plays a crucial role in cognitive functions like reading, comprehension, number processing, attention, reasoning, and social cognition. The thalamus collaborates with neocortical regions to select input of sensory information to the neocortex for processing in self-awareness, filtering out what is overwhelming or distracting [153]. As a component of the Default Mode Network, the angular gyrus is primarily focused on processing higher-level concepts creatively and manipulating mental representations in reasoning and social cognition [152, 154]. The angular gyrus also has a key role in production of vivid recollection of autobiographical memories, as well as multimodal integration of information during the encoding and retrieval of events in self-awareness [155].

The neuropsychological expression of integrated information depends on the reciprocal feedback interactions between the intrinsic and extrinsic systems for regulation of gene expression. These interactions are dependent upon a person’s meta-stable configuration of temperament and character, which in turn orchestrates adaptations to internal and external conditions that are dynamic, self-organizing, context-dependent, and idiographic. Parahippocampal processing of extrinsic sensory information is omni-modal when it enters memory for further information processing, but already pre-selected for attention by prior emotional state and temperament, which leads to differences between individuals in their unique patterns of sensory and emotional reactivity. Further processing, organization, and interpretation by intrinsic (top-down) brain connectivity networks is integrated in self-aware consciousness in the angular gyrus to influence what is meaningful by production of cross-modal symbols, metaphors, concepts, and figurative language. The pattern of these dynamic and self-organizing interactions is nevertheless meta-stable, like an individual’s personality, except at major tipping points when we encounter challenging situations that prompt increases or decreases in insight and judgment [69, 156].

Through increasing plasticity, complexity, and consciousness, human beings have acquired the ability to self-regulate and coordinate the expression and development of their own emotions, goals, and values to function coherently (i.e., in accord with one another) [69, 157]. However, the success of such integrative processes toward coherence varies widely between individuals in the extent to which it is healthy or unhealthy in terms of physical, mental, social, and spiritual well-being as a result of the complex interplay of diverse biopsychosocial influences [8, 64, 67, 68, 74, 157].

### Limitations and strengths

The major limitation of the project is that transcriptomic data was only available from one peripheral blood sample at one time in adulthood. This presented us with a challenge because of the complex nature of the pathway from the encrypted information stored in the genome that only unfolds over a person’s lifetime in different stages of development and under widely variable conditions. Fortunately, we were able to address this challenge with the innovative method we described in our introduction and methods sections. Access to systematic bioinformatic information allowed us to identify molecular connectivity networks involving latent interactions spanning a person’s lifetime.

The effectiveness of our approach is important to recognize because the complex adaptive systems related to human personality and related systems of learning and brain connectivity can never be understood from research on non-human experimental animals that lack the self-awareness and creativity that has allowed humans to transform life during the Anthropocene. Innovative approaches like what we applied here are essential for understanding human plasticity, complexity, and consciousness.

Fortunately, our use of colocalized expression in specific brain regions allows tests of hypotheses across multiple independent lines of evidence, including the evolution, structure, and functional dynamics of a reliably measured phenotype. Our findings confirmed our hypotheses, thereby replicating a consistent pattern of multi-modular information processing across the domains of phenomic, genomic, transcriptomic, and proteomic data.

### Conclusions and implications

We conclude that a control hub of six genes integrates two reciprocally interactive systems for regulation of personality-related gene expression in specific brain regions: an extrinsic and an intrinsic system, each with multiple specialized modules that turn one another on and off according to external and internal conditions. This coordinating hub is the crux of a molecular integrative network that orchestrates the information-transfer among its complex multi-modular system of over 4000 genes. The level of integration in the molecular integration network, and hence its coherence and efficiency, depended primarily on a person’s level of self-awareness (i.e., insight and judgment), as previously observed for brain functional connectivity [8].

The major functions of the genes in the regulatory networks we identified involve contributions to increasing plasticity, self-organized complexity, and consciousness by specific evolutionary mechanisms: the formation of membrane-less organelles in all life forms, diversity of gene-TF relationships in multicellular organisms, diversity of miRNAs in animals, and novel miRNAs-lncRNAs interactions in the neuronal systems of mammals, particularly hominids. Consequently, networks for gene regulation in self-aware humans are not only dynamically self-organized and context-dependent, but also health-promoting, open-ended, and creative. Human beings have the potential to function as “evolution aware of itself” [158], but vary widely in how well they are doing so under current world conditions [129].

## Supplementary information

Supplementary Information: Text

Supplementary Figure S1

Supplementary Figure S2

Supplementary Figure S3

Supplementary Figure S4

Supplementary Figure S5

Supplementary Figure S6

Supplementary Table S1

Supplementary Table S2

Supplementary Table S3

Supplementary Table S4

Supplementary Table S5

Supplementary Table S6

SupplementaryTable S7

Supplementary Table S8

Supplementary Table S9

Supplementary Table S10

Supplementary Table S11

Supplementary Table S12

Supplementary Table S13

Supplementary Table S14

Supplementary Table S15

Supplementary Table S16

Supplementary Table S17

Supplementary Table S18

Supplementary Table S19

Supplementa;ry Table S20

Supplementary Table S21

Supplementary Table S22

Supplementary Table S23

## Data Availability

The Young Finns Study granted data access to CRC and IZ by a Materials Transfer Agreement. The dataset supporting the conclusions of this article was obtained from the Young Finns Study which comprises health related participant data. The use of data is restricted under the regulations on professional secrecy (Act on the Openness of Government Activities, 612/1999) and on sensitive personal data (Personal Data Act, 523/1999, implementing the EU data protection directive 95/46/EC). Due to these restrictions, the data cannot be stored in public repositories or otherwise made publicly available. Data access may be permitted on a case-by-case basis upon request only. Data sharing outside the group is done in collaboration with YFS group and requires a data-sharing agreement. Investigators can submit an expression of interest to the chairman of the publication committee, Professor Mika Kähönen (Tampere University, Finland) and Professor Terho Lehtimäki (Tampere University, Finland).
