# Supplementary material for: Gene expression networks regulated by human personality
Source: Mol Psychiatry. 2024 Mar 4;29(7):2241–60. doi: 10.1038/s41380-024-02484-x (PMC11408262; doi:10.1038/s41380-024-02484-x)
Supplement: Supplementary file 1 — Supplementary Information: Text [file 41380_2024_2484_MOESM1_ESM.pdf]

Gene expression networks regulated by human personality

Supplementary Information

Outline

A. Text Sections

S1. Support for hypotheses about gene expression networks related to personality

S2. Methods for uncovering structure and function of TCMIN

S3. Analysis of differential gene expression by personality group and T-biclusters

S4. Demographics of sample by TC profile group

S5. Functional Annotation of brain regions with co-expressed T-GET genes

S6. Network analysis of genomic-environmental and transcriptomic genes

S7. Functional Annotation of Control Hub Genes for T-SAER-SASC

S8. A broader perspective on temperament-character integration

S9. Conservation of the six hub genes that regulate TCMIN

S10. Relation of TCMIN genes with previously described personality genes

B. Supplementary Figures

S1. T-SAER GET interaction analysis

S2. T-SASC GET interaction analysis

S3. T-SAER GET integration

S4. T-SASC GET integration

S5. Plasticity-related genes in TCMIN

S6. Orthologs of three miRNAs in TCMIN control hub across tree of life

C. Supplementary Tables

S1. Differential gene expression by group (ANOVA with HSD pairwise tests)

S2. Comparison of personality groups on demographics and body mass

S3. 728 genes in 7 transcriptomic-biclusters

S4. One-way ANOVA of differential gene expression among T-biclusters

S5. Pairwise t-tests of differential gene expression among T-biclusters

S6. Gene functions in transcriptomic biclusters T1 to T7

S7. Brain regions of interest with co-localized GE and T genes

S8. Brain region's mapping to GE and Transcriptomics sets

S9. Functions and related diseases of GET genes

S10. Differential specificity of T-SASC and T-SAER gene expression in various tissues

S11. TCMIN network nodes

S12. TCMIN genes (n = 4190), modules, and overlap with GETs

S13. Interactions of personality-related lncRNAs with 3 miRNAs in TCMIN control hub

(Gold shading indicates 7 lncRNAs unique to humans)

S14. Enrichment of LLPS-RNAs in 4376 genes of expanded TCMIN

S15. TCMI extended with genes connecting personality-related lncRNAs with TCMIN

(Red shading of name in column A indicates 13 ncRNAs corresponding to known personality related genes that were identified among 186 RNAs extending the TCMIN to total of 4376 genes. Pink shading in column G indicates the 129 RNAs that were known personality-related genes from GWAS and were identified in the original TCMIN with 4190 genes.)

S16. GO Cellular components associated with 3 miRNAs in TCMIN control hub

S17. Functional enrichment of TCMIN modules

S18. Phenotypes related to mutations in genes from TCMIN modules 1 - 4

S19. 4376 genes in extended TCMIN network for reviewed and high-throughput LLPS-RNAs by organelle subtype and associated control element

S20. Reviewed and high-throughput LLPS-RNAs genes found in TCMIN

S21. Chi-square tests for independence of LLPS-RNAs (reviewed and high-throughput)

S22. Orthologs of 3 miRNAs in TCMIN control hub

S23. Species in which all three miRNAs in TCMIN control hub are present

Supplementary Text Sections

S1. Prior support for hypotheses about gene expression networks related to human personality

Some of the personality-related genes we identified in GWAS are *regulatory genes* that influence the expression and co-expression of other genes. Most of the genes we found associated with human personality were transcribed to non-protein-coding RNAs, such as long-non-coding RNAs (lncRNAs) and microRNAs[1-5]. 95% of the 267 genes that were unique to modern humans, but not Neanderthals or chimpanzees, were transcribed to non-protein coding RNAs and 64% were in self-awareness genotypic system[5]. Instead of directly influencing their own transcription, these regulatory genes modulate the expression of downstream target genes[6]. SNPs in regulatory regions or in the coding sequence of regulatory genes can disrupt their function, leading to altered expression of their target genes without significant changes in their own transcription[6].

Personality-related genes identified by GWAS also include many genes involved in *signaling cascades* in response to external and internal stimuli. We found that genes for human temperament were enriched for Ras-MEK-ERK (MAPK), PI3K-AKT-mTOR, and Protein Kinase A, B, C pathways, which regulate associative conditioning as first messengers in response to extracellular stimuli[2, 4]. We found that the genes for intentional self-control aspects of character were enriched for the Phosphatidyl Inositol Calcium^{2+} signaling system that acts as an intracellular second messenger[1, 4]. Together these first and second messenger signaling pathways form cascades that transmit signals from cell surface receptors to the nucleus where they activate cellular processes underlying personality development, learning and memory, and the regulation of gene

expression[1-4, 7]. These gene products may act as intermediaries in transmitting signals, but their own expression levels may not be directly altered significantly by the personality-associated SNPs[8]. Nevertheless, their functional activity can still influence the expression of genes downstream in the signaling cascade. Bioinformatic tools are available to identify the genes that interact in networks that influence signaling pathways[9].

Although the majority of genes associated with human personality in GWAS are not protein coding, protein-coding genes constitute 48% of those associated with temperament[2] and 41% with character[1]. Thus the personality-related genes are likely to include translated proteins involved in large and complex *protein-protein interaction networks*[10]. Changes in the structure or function of these proteins due to SNPs can affect their interactions with other proteins, leading to cascading effects on the transcriptional activity of the interacting partners[11, 12]. This can result in transcriptomic changes in genes indirectly influenced by the SNPs related to personality that are identified by GWAS. Therefore, protein-protein interactions are expected to help to identify constituents of the interactive modules involved in regulating gene expression[13].

Personality-related SNPs identified by GWAS within one gene can also impact the epigenetic regulation of other genes, thereby participating in networks for epigenetic regulation[1, 5]. *Epigenetic modifications*, such as DNA methylation and histone modifications, play a crucial role in regulating gene expression[14]. Personality-related

SNPs within one gene for DNA methylation, transcription factor binding, and microRNA targets can disrupt the normal epigenetic landscape, leading to altered gene expression of neighboring or interacting genes without directly affecting their own transcription[15]. Prior work identified several character-related genes in GWAS that affect epigenetic processes of DNA methylation and chromatin modification[1, 4], so we reasonably expect to identify overlap between RNAs transcribed in whole blood with gene networks regulating epigenetic and chromatin modification.

Several personality-related SNPs in one gene identified by GWAS are involved in the regulation of *homeostasis and allostasis*. Homeostasis is the maintenance of physiological variables within healthy functional limits, and allostasis is the maintenance of stability in cellular and physiological functioning despite changing external and internal conditions[16, 17]. Some genes may indirectly alter the activity of other genes regulating gene expression to maintain stable cellular and physiological functioning of person[18, 19]. For example, 80% of p53 binding sites are co-localized with other TFs and epigenetic marks, which allow it to function as a dynamic regulator of context-dependent transcriptional networks that help to maintain the integrity of the genome and the functional stability of cells[19]. Likewise, personality can influence gene expression indirectly by context-dependent self-regulation of habits, goals, and values by interactions with other genes that automatically adapt flexibly to changes in self-regulated behavior[20]. Thus, we expect to identify an interactive network of genes

regulating homeostasis and allostasis that is influenced by human personality, particularly self-awareness as measured by the learning system indicated phenotypically by the creative-reliable personality profile and genotypically by the genomic network for creative self-awareness.

Finally, personality may enable the integration and orchestration of the multiple modules already described for transcriptional processes (e.g., transcription factor binding and epigenetic modification) and post-transcriptional processes (e.g., protein-protein interactions, microRNA-gene interactions) by means of reciprocal feedback interactions that allow these modules to turn one another on and off. Such integration and orchestration of a complex set of interactive modules is expected to underlie the open-ended development and evolution of life forms[21, 22], which may be adaptively regulated in at least some modern humans with self-control and creative self-awareness.

These prior observations led to some general hypotheses that we tested in this project. First, we hypothesize some aspects of gene expression and epigenetic change are regulated by reflexive-instinctive networks that change rapidly, reversibly, and automatically to maintain allostasis, and that the automatic regulation is only partially associated with an individual's self-reported temperament-character configuration. Rapid and reversible adaptation by automated responses is needed to maintain allostasis in response to changing conditions outside a person's self-control and self-

awareness. Consequently, allostatic regulation is not fully associated with human personality to the extent that some events and conditions that trigger change are independent of person and their temperament-character profile. It is well-established that the broad heritability of temperament and character is about 50% from GWAS and twin studies, and that most of the remaining variance is attributable to environmental events independent of the person and their family [1, 2, 4].

Second, we hypothesize that a person's temperament-character configuration maintains a meta-stable identity and outlook on life, which influences a person's initial perceptions[23] and, in turn, their gene expression and brain functional connectivity networks whether a person is awake, asleep, or anesthetized[24]. Put another way, personality and related networks of information-processing and gene expression are idiographic (specific, to a person's unique identity) and produce differences in a person's health, as previously documented[3, 18]. Therefore, we expect to find differences in gene expression associated with individuals with different temperament-character configurations.

Third, we have found that humans have three disjoint systems for learning and memory with multiple functional and structural modules for specialized information processing that need to be coordinated for healthy brain functioning[3, 18]. These different learning modules need to be integrated to bring their emotional reactivity and habits into accord with their goals and values to create and maintain a coherent identity and

life narrative[18, 25]. Therefore, we predict that regulation of gene expression in humans involves an efficient integrative network to coordinate multiple interactive modules of gene expression.

More specific molecular hypotheses are presented in the main text, but these general hypotheses are useful to consider our perspective on the regulation of gene expression networks in relation to human personality.

S2. Methods for uncovering structure and function of the Temperament-Character Molecular Integration Network (TCMIN)

TCMIN functional annotation: The functional annotation of the TCMIN gene modules, which were discovered through network analysis, was carried out using two approaches. One approach used automatic methods, such as Gene Ontology (GO)[26] and pathway enrichment analysis through the use of different resources such as g:Profiler[27] and Genecodis[28], as well as searching by their bibliography using VarElect[29]. The combined use of these sources enhanced the biological interpretation of the extensive gene lists, thereby facilitating the biological interpretation of the discovered modules. In the second approach, due to the limitations of gene ontology annotations (e.g., incomplete annotations, bias, limited scope and resolution), we did additional analysis based on the description of the gene functions using other available resources (e.g., WikiGene, Entrez, etc.) to identify concrete types of genes such as zinc fingers, histones, ubiquitins, or genes related to neuronal development and functioning.

Presence of RNAs involved in liquid–liquid phase separation in TCMIN: To explore the potential involvement of TCMIN genes in molecular processes that are hypothesized to be fundamental in the origin of cellular life and the evolution of the organization of intracellular functions, such as liquid-liquid phase separation (LLPS), the study utilized the RPS database (http://rps.renlab.org), a comprehensive repository of LLPS-related RNAs found in 20 distinct biomolecular condensates across eukaryotes and viruses. Additional gene ontology enrichment on the cellular component were carried out using the GO enrichment analysis tool (http://geneontology.org/docs/go-enrichment-analysis/ [26].

To determine the presence of significant differences between the LLPS genes found in the TCMIN and the human genome, a chi-square test for independence (i.e., non-enrichment) was employed. Subsequently, the chi-square statistic and associated p-value were calculated to evaluate the null hypothesis, which posits no significant difference in the distribution of LLPS genes between the two groups for reviewed and high-throughput LLPS data. The significance level was set at $\alpha = 0.05$. A result of "Reject H0" indicates a significant difference, whereas "Fail to reject H0" implies no significant difference (Supplementary Tables S14, S16, S21).

Evolution of the miRNAs that regulate the TCMIN: To examine the evolution of the regulation of intercellular functioning in multicellular organisms, evolutionary

homology relationships of the three most important miRNAs in the TCMIN were retrieved from available miRNA orthologs information using the Ensembl Comparative Genomics (Compara) API URL: http://www.ensembl.org .

Overlap of the TCMIN with genes unique to human personality and learning: We computed the overlapping genes between the TCMIN genes and the 972 personality-associated genes previously described by our group[3, 5]. Many of the genes unique to modern humans in the personality set were noncoding RNAs, including long-non-coding (lnc) RNAs and microRNAs (miRNAs)[5]. As previously explained, the TCMIN was constructed solely based on gene-miRNA and protein-protein interactions, so to investigate the potential associations of the TCMIN with lncRNA genes unique to modern humans, we searched for known interactions with these lncRNAs using the miRNet 2.0 network[30].

S3. Analysis of differential gene expression by personality profile group & T-biclusters

The expression data of 34,602 transcribed genes in blood samples from 459 individuals with creative-reliable, organized-reliable, or emotionally unregulated personality profiles were compared for differential gene expression. Bootstrap analysis of the subjects, where the expression of each of the 1500 genes were averaged for all individuals within the same network, indicated that the three networks are different

from each other (F=198.97, p<0, 0001, ANOVA, Tukey HSD test $p < 0.01$ for all pairwise combinations, after correction for multiple tests) (Table S1).

In addition, significant differences in the average expression of each gene were evaluated between T-biclusters (Supplementary Table S3). We performed an ANOVA test using the R function anova_test from the rstatix library (Supplementary Table S4). Moreover, pairwise analysis t-tests were performed between all possible combinations of biclusters for each gene by using pairwise t-test function and adjusting p-value method for multiple comparisons by Bonferroni from rstatix (R-package) (Supplementary Table S5).

S4. Demographics of sample by TC profile groups

We compared the personality profiles with one another to evaluate whether differences in demographics could account for the observed differential gene expression by TC profile groups (Supplementary Tables S2). Demographic variables, such as age and gender, and body mass index can influence gene expression profiles[31-33], so we examined these in our sample of Young Finns. For categorical vs numerical variables statistical results were calculated using ANOVA and t-tests (rstatix package, R). Results show an absence of strong effects from age and body mass index, as summarized in Supplementary Table S2. In each group, the average age was about 42 years, with non-significant ($p > 0.05$) and weak differences between groups (effect size $r = 0.001$). Similar

results were found for the body mass index (BMI) in kg/m2, in each group, the average BMI was about 26 kg/m2, with non-significant ($p > 0.05$) and weak differences between groups (effect size r = .0.004) (Supplementary Table S2). We used chi-square test for categorical vs categorical variables (chisq.test function from the R stats package).

Gender distribution was approximately 46% male and 54% female overall. The numbers of men and women were nearly equal in the organized-reliable and the emotional-unregulated profile groups, but the creative personality profile was more frequent in women than men (87 women vs 38 men). These gender differences are typical in western cultures where women usually mature in creative self-awareness earlier in childhood than men and then usually maintain this advantage until after age 60[34, 35].

The chi-square test of independence rejects the null hypothesis H0 and indicates that the row and the column variables are statistically significantly associated (p-value = 2.20E-04) (Supplementary Table S2). From the contribution results, the most contributing cells to the Chi-square are Creative/female (33.3%), Creative/male (39%). These cells contribute about 72% to the total Chi-square score and thus account for most of the difference between expected and observed values.

S5. Functional Annotation of brain regions with co-expressed T-GET genes

T-SAER and GET genes co-express in regions for self-regulation of anxiety: We found that the functions of the identified brain regions matched closely with the

functions of the corresponding networks for human personality and learning. Specifically, the brain regions in which transcripts were colocalized with genes associated with the interactions of the self-awareness and emotional reactivity networks (T-SAER GET) were involved in the self-regulation of anxiety (Figure 3A, Supplementary Table S9). The amygdala is located deep in the medial temporal lobe just anterior to the hippocampus and is involved in emotion regulation by its connectivity with the prefrontal and temporal cortices, the hypothalamus, and the cerebellum. The basomedial amygdala (BMA) is a key target of the ventral medial prefrontal cortex (vmPFC), which enables the differentiation of safe and aversive environments. This connection also facilitates the top-down control of anxiety and fear-related behavior, inducing long-lasting plasticity in response to stress[36]. The dentate nucleus is a vital structure located within the lateral cerebellar hemispheres, responsible for connecting the cerebellum to the rest of the brain. It is the most recent cerebellar nucleus to develop and has expanded significantly in humans. The dentate nucleus receives input from the cerebral cortex and plays a critical role in the planning, initiation, and control of voluntary movement, as well as conscious thought and visuospatial functions. Its function is essential for coordination of various processes, including thoughts and motor behavior, ensuring that they function smoothly and efficiently[37]. The parahippocampal gyrus is part of the limbic system; it receives and integrates sensory information from all the senses for emotion regulation, learning, and

memory in a dynamic biopsychosocial context. The middle temporal gyrus subserves language and semantic memory processing, visual perception, and multimodal sensory integration. Damage to these structures can result in both anterograde and retrograde amnesia, impairing new learning as well as memory for information acquired before the damage occurred[38]. The connections and functions of the basomedial amygdala and hippocampal-middle temporal regions interact to translate emotions, especially fear and anxiety, into outcomes adaptive to the person's self-awareness of their biopsychosocial context. The amygdala has the unusual role of screening and filtering its own input prior to hippocampal processing[39].

<u>T-SASC and GET are co-expressed in regions responsible for figurative language production:</u> We found that the T-SASC GET genes were co-localized in four brain regions involved in figurative language production: angular gyrus, lateral thalamic nuclei, cochlear nuclei, and middle temporal gyrus (Figure 3B). The angular gyrus processes multimodal sensory information such as somatosensory, auditory, and visual inputs. It plays a crucial role in cognitive functions like reading, comprehension, number processing, attention, reasoning, and social cognition. As a component of the Default Mode Network, it is primarily involved in processing higher-level concepts and manipulating mental representations[40]. The lateral thalamic nucleus which transmits limbic, sensory, and motor signals to cortex, regulates consciousness, sleep, and alertness. The cochlear nucleus is the first station for integrating and processing

auditory information bilaterally before sending it to other auditory centers. All four regions together are responsible for multimodal sensory processing, integration and interpretation of intellectual meaning, and dynamic mental representation for cross-modal symbols underlying metaphorical expression, language, and reading in self-aware consciousness[41].

S6. Network analysis of genomic-environmental and transcriptomic genes

To better understand the neurobiological relationships between the GE and the T genes we performed a network analysis at three different levels of biological interaction in the identified GET networks: gene-microRNA interactions, gene-transcription factor interactions, and gene-gene interactions for the T-SAER and T-SASC GET genes (Supplementary Figures S1, S2). These analyses enabled us to uncover the hidden minimum molecular network that linked all genes within each GET (Figure 3C, Supplementary Figures S3-S4). In other words, we found that the interaction of genetic-environmental and transcriptomic genes (GETs) creates small regulatory networks that function as interactive modules because they link functional genetic variation, environmental responses, transcriptional and post-transcriptional in brain, as described in the following subsections.

<u>The T-SAER GET molecular network coordinates positive and negative regulators of synaptic and cytoskeletal plasticity in response to sensory stimuli: </u>The

resulting T-SAER network was comprised of 45 genes (Figure 3C, Supplementary Figure S3 and Table S9). These included 10 out of the 11 seed GET genes, 20 transcription factors, 10 microRNAs and 4 protein-coding genes (Supplementary Figure S3 and Table S9). Functional annotation showed that 51% of these genes, including two zinc finger proteins (ZNF580 and ZNF341), were involved in positive or negative modulation of the cytoskeleton and/or synaptic plasticity. Migration and cytoskeleton-related processes are crucial for the morphological changes in cell structure that enable neuron plasticity. Genes related to synaptic plasticity were mainly involved in neural stem cell differentiation, neuron morphology, neuronal synapse formation and maturation, axon regrowth, and neuronal apoptosis (Supplementary Table S9). For example, the glycine receptor GLRA2 is widely distributed throughout the CNS, particularly within the hippocampus, spinal cord, and brain stem. GLRA2 is reported to have a novel role in synaptic plasticity, learning, and memory by linking altered glycinergic signaling to social and cognitive impairments[42].

The other 49% of the genes were mostly positive and negative regulators of epigenomic events (26%) and/or immune and stress responses consistent with their expected role in the interaction of self-awareness and emotional reactivity (Supplementary Table S9).

The T-SASC GET molecular network coordinates positive and negative regulation of epigenomic changes and inflammatory responses: The T-SASC network comprised 43 genes (Supplementary Figure S4 and Table S9). These included 13 seed GET genes, 19 transcription factors, 9 microRNAs and 3 protein coding genes (Figure S3). In contrast to findings for the T-SAER network, in the T-SASC network we found that most of the genes (44%) were involved in epigenetic events such as changes in the structure, function, and regulation of chromatin, and changes in gene expression through epigenetic changes (Supplementary Table S9).

21% of the 43 T-SASC genes were involved in processes related to plasticity. For example, TRIM28 controls the expression of transposable elements implicated in the regulation of human brain evolution and neurological disorders[43]. The remaining 35% of the genes in the T-SASC network involved glucose transport.

S7. Functional Annotation of Control Hub Genes for T-SAER-SASC

The protein-coding genes SSRP1 and UBC genes are related to epigenetic modification, including chromatin structure modification (SSRP1) and polyubiquitination (UBC), each also playing a role in DNA damage repair (Supplementary Table S9). SLC2A14 transports glucose across cell membranes and is important in development. All three, SLC2A14, SSRP1, and UBC, are involved in the regulation of gene expression and cellular metabolism. Mutations in SLC2A14 and UBC

have been linked to several psychiatric disorders (e.g., SLC2A14 to Alzheimer's disease) (Supplementary Table S9).

The three microRNAs (hsa-mir-1-3p, hsa-mir-34-5p, and hsa-mir-335-5p, are known to be associated with psychiatric and neurological disorders (Supplementary Table S9). Hsa-mir-1-3p is known to play a crucial role in a variety of developmental processes, particularly in the context of heart muscle[44] and brain development and function[45]. In the brain, hsa-miR-1-3p is involved in regulating neuronal differentiation, synaptic plasticity, and neurodegeneration, as described in the Introduction of the main article. It plays a critical role in the maturation of neurons and the formation of dendritic spines, as well as regulating the expression of genes involved in neurotransmitter release, synaptic function, neuroglial differentiation, dopaminergic neuron development, and nervous tissue myelination[46, 47]. Dysregulation of hsa-miR-1-3p has been implicated in neuropsychiatric disorders, including Alzheimer's disease, schizophrenia, and epilepsy (Supplementary Table S9)[45]. Antidepressant drugs have been shown to stimulate neurogenesis in the hippocampal brain region by stimulating the production and signaling of neurtrophins[48].

The microRNA hsa-mir-34-5p has been the subject of numerous studies in the fields of neurodevelopment, neuronal apoptosis, and cancer[49]. In these studies, it has been associated with the repression of genes involved in synaptic plasticity, such as

brain-derived neurotrophic factor (BDNF) and SIRT1, which are important for learning and memory[50]. It also plays a role in energy metabolism, and resting state network activity[51]. In addition, miR-34a-5p has been implicated in the pathogenesis of several neurological disorders, including Alzheimer's disease and Parkinson's disease. In Alzheimer's disease, miR-34a-5p has been found to contribute to beta-amyloid-induced neurotoxicity, whereas in Parkinson's disease, it has been shown to regulate the expression of genes involved in the functioning of dopaminergic neurons[52] (Supplementary Table S9).

The microRNA hsa-mir-335-5p has been shown to orchestrate various cellular processes in the brain, including neurogenesis, synaptic plasticity, and neuronal differentiation. Its inhibition triggers changes in mitochondrial physiology and increases apoptosis[53]. In Alzheimer's disease, it has been found to regulate the expression of genes involved in amyloid-beta metabolism[54]. In Parkinson's disease, it regulates the expression of genes involved in dopaminergic neuron survival[55]. In epilepsy, it plays a role in regulating the expression of genes involved in neuronal excitability[56].

S8. A broader perspective on temperament-character integration

The uncovered T-SASC-SAER network revealed how functional genomic variation in personality and learning is integrated with transcriptional plasticity in a person's

brain. The plasticity occurs in response to changes in external and internal stimuli, including extracellular stimuli, cellular and organismal stressors, and internal processing of the meaning of events and symbols. These observations confirmed our hypothesis that human personality depends on complex adaptive process involving multiple biopsychosocial systems of learning and memory that must be integrated for healthy functioning. These complex and dynamic processes must consider the nature and strength of the stimuli and mechanisms for adaptation to changing environments with flexible transcriptional processes. Thus, to uncover the broader underlying regulatory network that we had hypothesized, we examined all interactions at both miRNA-gene and gene-gene levels for the six central genes (SLC2A14, SSRP1, UBC, hsa-mir-1-3p, hsa-mir-335-5p, and hsa-mir-34a-5p) that coordinate the integrated genomic – environmental – transcriptional network.

Our bioinformatic findings revealed that these six coordinating genes directly interact with 4190 genes, including 3919 protein-coding genes, 198 microRNAs, 38 pseudogenes, and 35 other ncRNAs. These 4190 genes are organized as 10 functional modules (Figure 4A), including regulation of nervous system development (M1), response to sensory stimuli (M2), chromosome and organelle organization (M3), axogenesis (M4), regulation of lipid levels, FZD ubiquitination, and extracellular matrix organization (M5), chromatin organization and transcription regulation (M6), hemostasis, membrane trafficking, and intercellular communication (M7), and clusters

of microRNAs that interact with the three protein-coding genes SLC2A14 (M8), UBC (M9), and SSRP1 (M10). The interaction of these groups of genes gives rise to the temperament-character molecular integration network (TCMIN) displayed in Figure 4A and characterized in more detail in Supplementary Tables S11 and S12.

To evaluate the role of interactions of the microRNAs in TCMIN more fully, their known interactions with personality-related ncRNAs were added, extending the number of genes in TCMIN to 4376 (Figure 4B, Supplementary Tables S14 and S13). These genes in the extended TCMIN included 3919 protein-coding genes, 371 microRNAs, 38 pseudogenes, and 48 other ncRNAs. 129 of the 972 personality-associated genes from our prior GWAS were recovered in the expanded TCMIN (Supplementary Table S15). To describe the functional organization of the molecular integration network, these modules are described systematically in the following subsections.

<u>MicroRNAs are key control elements of the TCMIN network:</u> Network analysis revealed that hsa-mir-1-3p, hsa-mir-335-5p, and hsa-mir-34a-5p are important hubs of the TCMIN. These three miRNAs interacted with hundreds of protein genes either individually, in pairs or all three together (Figure 4A).

The 2413 genes in the module of genes that interacted uniquely with microRNA hsa-mir-335-5p (Module 5 in Figure 4A, Supplementary Tables S11, S17) are involved in

the regulation and maintenance of cellular structure and function, including the control of lipid levels (e.g., cholesterol), the modification of key signaling receptors (e.g. FZD ubiquitination), and extracellular matrix organization (Supplementary Table S17). The module of genes interacting only with microRNA hsa-mir-34a-5p is comprised of 577 genes mostly involved in regulation and maintenance of gene expression during development, including the control of chromatin structure, the transcriptional activation or repression of genes, and the orchestration of complex developmental biological processes (Module 6 in Figure 4A, Tables S11, S17).

The module of 708 genes that interacts only with microRNA hsa-mir-1-3p regulates and maintains cellular and physiological processes, including hemostasis, membrane trafficking, and cell-cell communication (Module 7 in Figure 4A, Supplementary Tables S11, S17).

The three hub microRNAs act in coordinated manner over specific sets of genes: The microRNAs, hsa-mir-1-3p, hsa-mir-335-5p, and hsa-mir-34a-5p, also control smaller, more specific groups of genes in pairs or altogether. Module 1 of the TCMIN network (Figure 4A) is comprised 122 protein-coding genes modulated jointly by hsa-mir-1-3p and hsa-mir-335-5p. The genes in Module 1 were enriched for genes involved in biological processes for nervous system development, especially axogenesis and neurogenesis (Supplementary Table S17). Variants in these genes are related to

neuropsychiatric disorders (Supplementary Table S18); for example, SH3TC2 is expressed in Schwann cells and is necessary for proper myelination of peripheral axons[57].

Module 2 of TCMIN is comprised of 80 protein-coding genes modulated jointly by microRNAs hsa-mir-34-5p and hsa-mir-335-5p. It was enriched for genes coding for sensory responses to diverse types of stimuli, including mechanical stimuli, gravity, oxidative stress, hormones, or various chemical substances) (Supplementary Table S17). Variants of all genes in this group have been linked to phenotypes involved in modifications of stimulus responses and neuronal activity in brain (Supplementary Table S18). For example, SYT1 is a stimulus-dependent gene that regulates both the rate and the size of synaptic vesicles formed during endocytosis; it is also required during exocytosis[58].

Module 3 is comprised of 67 protein-coding genes modulated jointly by microRNAs hsa-mir-1-3p and hsa-mir-34-5. The M3 group was enriched only in GO terms related to cellular component organization regulation (Supplementary Table S17). All genes in this group were also related to phenotypes involved neuronal development and psychiatric anomalies or chromatin and organelle organization, such as SMARCC1 that stimulates the remodeling activity of nucleosomes[59] (Supplementary Table S18).

Module 4 of TCMIN was the only module regulated by all three microRNAs of the central hub. It was comprised of 8 protein-coding genes that were enriched in GO axogenesis (Supplementary Table S17). Variants in these genes are documented to present phenotypes that alter *axonal* growth, fasciculation, and synaptogenesis (Supplementary Table S18). One of the most representative genes in this group is L1CAM, which plays a crucial role in the formation of major axonal tracts such as the corticospinal tract and corpus callosum[60].

Protein-coding genes SLC2A14, UBC and SSRP1 interact with small clusters of microRNAs: The three protein-coding genes in the molecular integration network interact with Modules 8, 9 and 10. Especially interesting is Module 10 (M10-SSRP1) which consists of 69 microRNAs coordinated by SSRP1. This module includes six miRNA members of the hsa-let7 family (hsa-let-7c-5p, hsa-let-7c-5p, hsa-let-7a-5p, hsa-let-7g-5p, hsa-let-7f-5p, and hsa-let-7i-5p). Studies have shown that hsa-let-7c-5p and hsa-let-7b-5p play important roles in regulating neural differentiation and development. Specifically, hsa-let-7c-5p has been shown to influence neuronal differentiation, neuronal subtype specification and synapse formation in animal models. Hsa-let-7b-5p has been shown to mediate regulatory epigenetic functions that influence the development and activity of human induced neuronal cells (iNs). However, overexpression of hsa-let-7c in human iNs can lead to morphological and functional deficits, including impaired neuronal morphological development, synapse formation

and strength, and reduced neuronal excitability. In addition, hsa-let-7b-5p has been found to regulate osteogenic differentiation in some human stem cells. The other members of the family have been described to be aberrantly expressed in major depression (hsa-let-7f-5p, hsa-let-7g-5p), Alzheimer's disease (hsa-let-7g-5p), bipolar disorder (hsa-let-7f-5p) and sporadic Creutzfeldt-Jakob disease (hsa-let-7i-5p).

The three protein-coding genes in the TCMIN central hub also interact with another individual microRNA, has-mir-29a-3p, which has been found to control innate and adaptive immune responses to intracellular bacterial infection by targeting interferon-Y[61].

Presence of many plasticity-related genes in TCMIN: Our manual curation process revealed many Zinc finger genes (n=143) in the TCMIN (Supplementary Figure S5, Table S12), which are related to the development, maintenance, and functioning of neuronal cells. Zinc fingers have been found to play a vital role in neural development, synaptic plasticity, learning and memory, and in the regulation of neurotransmitter receptors, ion channels, and other signaling molecules that are essential for proper brain function. Specifically, in the TCMIN genes, these Zinc fingers were primarily located in the clusters M1 (hsa-mir-1-3p and hsa-mir-335-5p), M2 (hsa-mir-34-5p and hsa-mir-335-5p), M3 (hsa-mir-1-3p and hsa-mir-34-5), M5 (hsa-mir-335-1p only), and M7 (hsa-mir-1-3p only). It is noteworthy that the M1 and M2 groups (Figure 4A) were enriched in genes involved in regulating the nervous system and stimuli response. In addition, the

TCMIN network contains another 5 genes that encode nuclear proteins that contain a zinc finger-like PHD (i.e., plant homeodomain) finger distinct from other classes of zinc finger motifs). These PHD fingers are involved in chromatin-mediated gene regulation (PHD finger).

Moreover, histones (n=61) and ubiquitins (n=40) (Supplementary Table S12) were exclusively present in the M6 (hsa-mir-34-5p only) and the M3 (hsa-mir-34-5p together with has-mir-1-3p) clusters (Figure 4A). These clusters are involved in chromatin organization (M6) or chromosome and organelle organization (M3) (Table S6). Additionally, the manual curation analysis identified 49 genes associated with neuronal system development, 25 genes related to synapse, and 38 pseudogenes (Supplementary Table S12).

<u>TCMIN genes act in a dynamic and coordinated way</u>: The TCMIN genes exhibit a noteworthy enrichment in genes associated with liquid-liquid phase separation (LLPS). This process enables specific proteins and RNAs to create membrane-less organelles (MLOs). This finding is supported by chi-square tests conducted at a significance level of $\alpha = 0.05$, yielding a p-value=1.452724e-09 for reviewed LLPS-RNA; and a p-value= 0.0E+00 for high-throughput LLPS-RNA (Supplementary Tables S14, S19-21, S15 - S17). Genes in TCMIN included 32% of the 72 previously known LLPS-RNAs in the comprehensive RPS database (Supplementary Tables S14). This is striking

when compared to the percentage of such LLPS-RNAs in the entire human genome (0.12 %, Supplementary Table S14). The enrichment is even more significant when based on the high-throughput RPS database of 9571 LLPS-RNAs: The TCMIN contains 1699 high-throughput LLPS-RNA genes, so 39% of the genes in TCMIN are related to LLPS compared to 15.7% of the total human genome (Supplementary Table S14).

The genes directly regulated by the three microRNAs in the hub of TCMIN include many related to LLPS. 51% of the genes regulated by microRNA has-mir-1-3p are high-throughput LLPS-related RNAs, as are 37% to 39% of the genes regulated by the other two hub microRNAs. More than half of the genes in functional modules regulated by multiple hub microRNAs are high-throughput LLPS-related RNAs: M1 for regulation of nervous system development with 59.0%, M2 for stimulus response with 57.5%, M3 for chromosome and organelle organization with 52.9%, and M4 for axonogenesis with 62.5% (Figure 4, Supplementary Tables S19, S20).

There are 12 different types of membrane-less organelles (MLOs) described in the RPS database. Stress granules (SG) were the most common MLOs related to the TCMIN's genes, representing 0.18% of the total found, followed by mesh-like networks (0.14%) and liquid droplets (0.115%) (see Supplementary Table S14). These findings were also supported by the Cellular Components Gene Ontology enrichment results, which show a significant enrichment in genes located in different types of membranes

such as vesicles, vacuoles, organelles, endo-lysosomes, and secretory granules (Supplementary Table S16).

Functional annotation of TCMIN genes indicates that they are involved in the coordination of many important processes, suggesting a regulatory mechanism of the network that might allow genes to act in a coordinated manner. An important biological process that enables this coordination is liquid-liquid phase separation (LLPS), which allows certain proteins and RNAs to form membrane-less droplets in the cytoplasm or nucleoplasm. These droplets act as MLOs that compartmentalize and coordinate biochemical reactions, regulate gene expression, and participate in signaling. A key advantage of LLPS-based regulation is that it enables rapid and reversible assembly and disassembly of MLOs in response to changes. This allows cells to rapidly adapt to changing environmental or physiological conditions and regulate their functions in a precise and controlled manner. MLOs have been shown to be essential for regulating stress responses[62], maintaining homeostasis[63], and facilitating development[64]. Dysregulation of LLPS is closely associated with several diseases[65, 66].

S9. Conservation of the six hub genes that regulate TCMIN

The TCMIN analysis demonstrated that the microRNAs hsa-mir-1-3p, hsa-mir-335-5p, and hsa-mir-34a-5p were highly important nodes in the network (Figure 4A, Supplementary Table S12), regulating specific sets of genes individually and

collaborating in pairs or together to control smaller, more specific groups of genes. To investigate the evolutionary conservation of these microRNAs, we extracted orthologs from 151 genomes across the tree of life (Supplementary Figure S6). Our results revealed that hsa-mir-34a-5p was the most conserved microRNA, with orthologs present in 96 different genomes, 89 of which were 1-to-1 orthologs (Supplementary Table S22). Hsa-mir-1-3p was present in 93 different genomes, with 84 1-to-1 orthologs (Supplementary Table S22), while hsa-mir-335-5p was the least conserved, with 85 orthologs, 82 of which were 1-to-1 orthologs in different genomes (Supplementary Table S22). However, it is interesting the fact that hsa-mir-335-5p was the only miRNA found in many Mammalia, from Primates such as the old-world monkey to alpaca or rabbits. Among all three miRNAs, has-mir-1-3pwas the one present in a wider spectrum of the tree life including some birds, marsupials and one reptile.

It is interesting that all three microRNAs were only found together in 28 mammalian genomes (Supplementary Table S23). There was variation in the percentage of sequence similarity between the query and target in all cases, except for identical sequences in three hominid genomes (chimpanzees, bonobos, and gorillas). Of these 28 genomes, eight were primates, including three of the suborder hominidae *(Pan troglodytes, Pan paniscus*, and *Gorilla gorilla gorilla*), three of the suborder Similiformes (*Chlorocebus sabaeus, Macaca fascicularis*, and *Macaca mulatta*), and one of suborder Strepsirhini (*Microcebus murinus*). Only one Feliforme genome (*Panthera pardus*)

appeared, along with six Artiodactyla genomes, eight Rodentia genomes from different suborders, two Carnivora genomes from different Canidae suborders, two Perissodactyla genomes, and one Proboscidea genome from the Elephantidae suborder.

Among the 3 hub protein-coding genes, Solute-Carrier Family 2, Member 14 (SLC2A14) is a highly conserved integral transmembrane protein that facilitates transport of hexoses, such as glucose and fructose, into all mammalian cells (NCBI gene summary in Gene Card). Ubiquitin C (UBC) is also a highly conserved gene that encodes an enzyme that facilitates protein binding. Structure Specific Recognition Protein 1 (SSRP1) has a highly conserved core domain, but varies in its other domains, which allows a variety of functions related to its roles in chromatin organization and binding with RNA, DNA, protein, and nucleosome binding and chromatin organization.

S10. Relation of TCMIN genes with previously described personality genes

When we compared the 4190 genes from the TCMIN with personality-associated genes identified and replicated in our prior work[3], we found that the three hub-miRNAs from the TCMIN coordinate 142 genes from the 972 personality genes. It is noteworthy that we identified six "switch genes" (BMP7, NR3C2, RGS13, VPS8, ZNF503, and SLC44A5) among them (Figure 4B). These switch genes were previously described as genes related to changes in health status among individuals with similar character

profiles[1]. BMP7 is a ligand of TGF-beta that supports the development of the cortex through the CSF and meninges[67]. Variations in the NR3C2 gene have been linked to differences in personality traits such as neuroticism and extraversion[68]. VPS8 is crucial for maintaining cell homeostasis and the proper functioning of the endosomal system, and alterations in this system have been associated with neurological diseases[69]. SLC44A5, although its function has not been clearly elucidated, is from the choline membrane transporter family which are also precursors of acetylcholine, a neurotransmitter that regulates various functions in the body, including stress-related responses[70]. The functions of RGS13 and ZNF503 in relation to the brain have not been elucidated yet. Except for BMP7, all other 5 switch genes are in the cluster regulated by hsa-mir-335-5p (Module 5 as shown in Figure 4B and Supplementary Table S12).

Because TCMIN was constructed solely based on gene-miRNA, TF-gene, and protein-protein interactions we further investigated the relation of TCMIN with personality-related lncRNA genes. Personality-associated lncRNAs are of special interest because some of them are unique to modern humans[5]. The results revealed a total of 102 interactions involving 20 long non-coding RNAs, seven of which are unique to humans (CASC15, LINC00472, LINC01450, LMCD1-AS1, UGDH-AS1, ZNF503-AS1, and ZNF571-AS1) (Supplementary Figure S5, Table S13). Additionally, 61 miRNAs from personality genes were found to interact with the TCMIN. These interactions were facilitated by small clusters interacting with SLC2A14, UBC, and SSRP1 (Supplementary

Figure S5). UBC is the major contributor to the ubiquitin RNA pool and is upregulated upon proteasome inhibition and oxidative stress. SSRP1 plays a role in DNA repair, gene transcription, and gene activation while SLC2A14 is a highly conserved integral membrane protein that transports hexoses such as glucose and fructose into all mammalian cells and has been associated with several chronic diseases.

References

1. Zwir I, Arnedo J, Del-Val C, Pulkki-Raback L, Konte B, Yang SS *et al.* Uncovering the complex genetics of human character. *Mol Psychiatry* 2020; **25**(10)**:** 2295-2312.

2. Zwir I, Arnedo J, Del-Val C, Pulkki-Raback L, Konte B, Yang SS *et al.* Uncovering the complex genetics of human temperament. *Mol Psychiatry* 2020; **25**(10)**:** 2275-2294.

3. Zwir I, Arnedo J, Del-Val C, Pulkki-Råback L, Konte B, Yang SS *et al.* Three genetic-environmental networks for human personality. *Molecular Psychiatry* 2021; **26**(8)**:** 3858-3875.

4. Cloninger CR, Zwir I. Genetics of human character and temperament. In: Cooper DN (ed). *Encyclopedia of Life Sciences (eLS)*, vol. 3. John Wiley & Sons, Ltd.: Chichester, U.K., 2022, pp 1-24.

5. Zwir I, Del-Val C, Hintsanen M, Cloninger KM, Romero-Zaliz R, Mesa A *et al.* Evolution of genetic networks for human creativity. *Mol Psychiatry* 2022; **27**(1)**:** 354-376.

6. Engreitz JM, Haines JE, Perez EM, Munson G, Chen J, Kane M *et al.* Local regulation of gene expression by lncRNA promoters, transcription and splicing. *Nature* 2016; **539**(7629)**:** 452-455.

7. Cloninger CR, Cloninger KM, Zwir I, Keltikangas-Jarvinen L. The complex genetics and biology of human temperament: a review of traditional concepts in relation to new molecular findings. *Transl Psychiatry* 2019; **9**(1)**:** 290.

8. Shastry BS. SNPs: impact on gene function and phenotype. *Methods Mol Biol* 2009; **578:** 3-22.

9. Cirillo E, Parnell LD, Evelo CT. A Review of Pathway-Based Analysis Tools That Visualize Genetic Variants. *Front Genet* 2017; **8:** 174.

10. Nibbe RK, Chowdhury SA, Koyuturk M, Ewing R, Chance MR. Protein-protein interaction networks and subnetworks in the biology of disease. *Wiley Interdiscip Rev Syst Biol Med* 2011; **3**(3)**:** 357-367.

11. Zhao N, Han JG, Shyu CR, Korkin D. Determining effects of non-synonymous SNPs on protein-protein interactions using supervised and semi-supervised learning. *PLoS Comput Biol* 2014; **10**(5)**:** e1003592.

12. Shatoff E, Bundschuh R. Single nucleotide polymorphisms affect RNA-protein interactions at a distance through modulation of RNA secondary structures. *PLoS Comput Biol* 2020; **16**(5)**:** e1007852.

13. Yang Y, Peng X, Ying P, Tian J, Li J, Ke J *et al.* AWESOME: a database of SNPs that affect protein post-translational modifications. *Nucleic Acids Res* 2019; **47**(D1)**:** D874-D880.

14. Portela A, Esteller M. Epigenetic modifications and human disease. *Nat Biotechnol* 2010; **28**(10)**:** 1057-1068.

15. Vohra M, Sharma AR, Prabhu BN, Rai PS. SNPs in Sites for DNA Methylation, Transcription Factor Binding, and miRNA Targets Leading to Allele-Specific Gene Expression and Contributing to Complex Disease Risk: A Systematic Review. *Public Health Genomics* 2020; **23**(5-6)**:** 155-170.

16. Sterling P. Allostasis: a model of predictive regulation. *Physiol Behav* 2012; **106**(1)**:** 5-15.

17. Schulkin J, Sterling P. Allostasis: A Brain-Centered, Predictive Mode of Physiological Regulation. *Trends Neurosci* 2019; **42**(10)**:** 740-752.

18. Zwir I, Arnedo J, Mesa A, Del Val C, de Erausquin GA, Cloninger CR. Temperament & Character account for brain functional connectivity at rest: A

diathesis-stress model of functional dysregulation in psychosis. *Mol Psychiatry* 2023; **https://doi.org/10.1038/s41380-023-02039-6**.

19. Nguyen TT, Grimm SA, Bushel PR, Li J, Li Y, Bennett BD *et al.* Revealing a human p53 universe. *Nucleic Acids Res* 2018; **46**(16)**:** 8153-8167.

20. Cloninger CR, Appleyard J, Mezzich JE, Salloum I, Snaedal J. Health is a creative adaptive process: Implications for improving health care and reducing burn-out around the world. *World Medical Journal* 2021; **67**(3)**:** 2-13.

21. Kitano H. Computational systems biology. *Nature* 2002; **420**(6912)**:** 206-210.

22. Davies P. What is Life? *New Scientist* 2019; **241**(3215)**:** 28-31.

23. Cloninger CR. Temperament and personality. *Curr Opin Neurobiol* 1994; **4**(2)**:** 266-273.

24. Zwir I, Arnedo J, Mesa A, del Val C, de Erausquin GA. Personality accounts for resting-state functional connectivity in psychosis: A biopsychosocial model of human brain functins. *Molecular Psychiatry* in review.

25. Garcia D, Cloninger KM, Cloninger CR. Coherence of character and temperament drives personality change toward well being in person-centered therapy. *Current Opinion in Psychiatry* 2023; **36**(1)**:** 60-66.

26. Ashburner M, Ball CA, Blake JA, Botstein D, Butler H, Cherry JM *et al.* Gene ontology: tool for the unification of biology. The Gene Ontology Consortium. *Nat Genet* 2000; **25**(1)**:** 25-29.

27. Raudvere U, Kolberg L, Kuzmin I, Arak T, Adler P, Peterson H *et al.* g:Profiler: a web server for functional enrichment analysis and conversions of gene lists (2019 update). *Nucleic Acids Res* 2019; **47**(W1)**:** W191-W198.

28. Garcia-Moreno A, Lopez-Dominguez R, Villatoro-Garcia JA, Ramirez-Mena A, Aparicio-Puerta E, Hackenberg M *et al.* Functional Enrichment Analysis of Regulatory Elements. *Biomedicines* 2022; **10**(3).

29. Stelzer G, Plaschkes I, Oz-Levi D, Alkelai A, Olender T, Zimmerman S *et al.* VarElect: the phenotype-based variation prioritizer of the GeneCards Suite. *BMC Genomics* 2016; **17 Suppl 2**(Suppl 2)**:** 444.

30. Chang L, Zhou G, Soufan O, Xia J. miRNet 2.0: network-based visual analytics for miRNA functional analysis and systems biology. *Nucleic Acids Res* 2020; **48**(W1)**:** W244-W251.

31. Vinuela A, Brown AA, Buil A, Tsai PC, Davies MN, Bell JT *et al.* Age-dependent changes in mean and variance of gene expression across tissues in a twin cohort. *Hum Mol Genet* 2018; **27**(4)**:** 732-741.

32. Oliva M, Munoz-Aguirre M, Kim-Hellmuth S, Wucher V, Gewirtz ADH, Cotter DJ *et al.* The impact of sex on gene expression across human tissues. *Science* 2020; **369**(6509).

33. Joseph PV, Jaime-Lara RB, Wang Y, Xiang L, Henderson WA. Comprehensive and Systematic Analysis of Gene Expression Patterns Associated with Body Mass Index. *Sci Rep* 2019; **9**(1)**:** 7447.

34. Zohar AH, Zwir I, Wang J, Cloninger CR, Anokhin AP. The development of temperament and character during adolescence: The processes and phases of change. *Dev Psychopathol* 2019; **31**(2)**:** 601-617.

35. Cloninger CR. Completing the psychobiological architecture of human personality development: Temperament, character, and coherence. In: Staudinger UM, Lindenberger UER (eds). *Understanding human development: Dialogues with lifespan psychology*. Kluwer Academic Publishers: London, 2003, pp 159-182.

36. Adhikari A, Lerner TN, Finkelstein J, Pak S, Jennings JH, Davidson TJ *et al.* Basomedial amygdala mediates top-down control of anxiety and fear. *Nature* 2015; **527**(7577)**:** 179-185.

37. Saab CY, Willis WD. The cerebellum: organization, functions and its role in nociception. *Brain Res Brain Res Rev* 2003; **42**(1)**:** 85-95.

38. Bayley PJ, Hopkins RO, Squire LR. The fate of old memories after medial temporal lobe damage. *J Neurosci* 2006; **26**(51)**:** 13311-13317.

39. Nauta WJH, Feirtag M. *Fundamental Neuroanatomy*. W.H. Freeman & Co: New York, 1986.

40. Seghier ML. The angular gyrus: multiple functions and multiple subdivisions. *Neuroscientist* 2013; **19**(1)**:** 43-61.

41. Benedek M, Beaty R, Jauk E, Koschutnig K, Fink A, Silvia PJ *et al.* Creating metaphors: the neural basis of figurative language production. *Neuroimage* 2014; **90**(100)**:** 99-106.

42. Pilorge M, Fassier C, Le Corronc H, Potey A, Bai J, De Gois S *et al.* Genetic and functional analyses demonstrate a role for abnormal glycinergic signaling in autism. *Mol Psychiatry* 2016; **21**(7)**:** 936-945.

43. Grassi DA, Jonsson ME, Brattas PL, Jakobsson J. TRIM28 and the control of transposable elements in the brain. *Brain Res* 2019; **1705:** 43-47.

44. Zhao Y, Ransom JF, Li A, Vedantham V, von Drehle M, Muth AN *et al.* Dysregulation of cardiogenesis, cardiac conduction, and cell cycle in mice lacking miRNA-1-2. *Cell* 2007; **129**(2)**:** 303-317.

45. Coccia E, Masanas M, Lopez-Soriano J, Segura MF, Comella JX, Perez-Garcia MJ. FAIM Is Regulated by MiR-206, MiR-1-3p and MiR-133b. *Front Cell Dev Biol* 2020; **8:** 584606.

46. Hollins SL, Goldie BJ, Carroll AP, Mason EA, Walker FR, Eyles DW *et al.* Ontogeny of small RNA in the regulation of mammalian brain development. *BMC Genomics* 2014; **15**(1)**:** 777.

47. Li P, Li Y, Dai Y, Wang B, Li L, Jiang B *et al.* The LncRNA H19/miR-1-3p/CCL2 axis modulates lipopolysaccharide (LPS) stimulation-induced normal human astrocyte proliferation and activation. *Cytokine* 2020; **131:** 155106.

48. Castren E, Voikar V, Rantamaki T. Role of neurotrophic factors in depression. *Curr Opin Pharmacol* 2007; **7**(1)**:** 18-21.

49. Jauhari A, Yadav S. MiR-34 and MiR-200: Regulator of Cell Fate Plasticity and Neural Development. *Neuromolecular Med* 2019; **21**(2)**:** 97-109.

50. Shi J. Regulatory networks between neurotrophins and miRNAs in brain diseases and cancers. *Acta Pharmacol Sin* 2015; **36**(2)**:** 149-157.

51. Sarkar S, Jun S, Rellick S, Quintana DD, Cavendish JZ, Simpkins JW. Expression of microRNA-34a in Alzheimer's disease brain targets genes linked to synaptic plasticity, energy metabolism, and resting state network activity. *Brain Res* 2016; **1646:** 139-151.

52. Segaran RC, Chan LY, Wang H, Sethi G, Tang FR. Neuronal Development-Related miRNAs as Biomarkers for Alzheimer's Disease, Depression, Schizophrenia and Ionizing Radiation Exposure. *Curr Med Chem* 2021; **28**(1)**:** 19-52.

53. De Luna N, Turon-Sans J, Cortes-Vicente E, Carrasco-Rozas A, Illan-Gala I, Dols-Icardo O *et al.* Downregulation of miR-335-5P in Amyotrophic Lateral Sclerosis Can Contribute to Neuronal Mitochondrial Dysfunction and Apoptosis. *Sci Rep* 2020; **10**(1)**:** 4308.

54. Wang D, Fei Z, Luo S, Wang H. MiR-335-5p Inhibits beta-Amyloid (Abeta) Accumulation to Attenuate Cognitive Deficits Through Targeting c-jun-N-terminal Kinase 3 in Alzheimer's Disease. *Curr Neurovasc Res* 2020; **17**(1)**:** 93-101.

55. Oliveira SR, Dionisio PA, Gaspar MM, Correia Guedes L, Coelho M, Rosa MM *et al.* miR-335 Targets LRRK2 and Mitigates Inflammation in Parkinson's Disease. *Front Cell Dev Biol* 2021; **9:** 661461.

56. Heiland M, Connolly NMC, Nguyen NT, Kesavan JC, Fanning K, Sanfeliu A *et al.* MicroRNA-335-5p suppresses voltage-gated sodium channel expression and may be a target for seizure control. *BioRXiv* 2022.

57. Gouttenoire EA, Lupo V, Calpena E, Bartesaghi L, Schupfer F, Medard JJ *et al.* Sh3tc2 deficiency affects neuregulin-1/ErbB signaling. *Glia* 2013; **61**(7)**:** 1041-1051.

58. Poskanzer KE, Fetter RD, Davis GW. Discrete residues in the c(2)b domain of synaptotagmin I independently specify endocytic rate and synaptic vesicle size. *Neuron* 2006; **50**(1)**:** 49-62.

59. Phelan ML, Sif S, Narlikar GJ, Kingston RE. Reconstitution of a core chromatin remodeling complex from SWI/SNF subunits. *Mol Cell* 1999; **3**(2)**:** 247-253.

60. Kamiguchi H, Lemmon V. A neuronal form of the cell adhesion molecule L1 contains a tyrosine-based signal required for sorting to the axonal growth cone. *J Neurosci* 1998; **18**(10)**:** 3749-3756.

61. Ma F, Xu S, Liu X, Zhang Q, Xu X, Liu M *et al.* The microRNA miR-29 controls innate and adaptive immune responses to intracellular bacterial infection by targeting interferon-gamma. *Nat Immunol* 2011; **12**(9)**:** 861-869.

62. Khong A, Matheny T, Jain S, Mitchell SF, Wheeler JR, Parker R. The Stress Granule Transcriptome Reveals Principles of mRNA Accumulation in Stress Granules. *Mol Cell* 2017; **68**(4)**:** 808-820 e805.

63. Audas TE, Jacob MD, Lee S. Immobilization of proteins in the nucleolus by ribosomal intergenic spacer noncoding RNA. *Mol Cell* 2012; **45**(2)**:** 147-157.

64. Zhang B, Arun G, Mao YS, Lazar Z, Hung G, Bhattacharjee G *et al.* The lncRNA Malat1 is dispensable for mouse development but its transcription plays a cis-regulatory role in the adult. *Cell Rep* 2012; **2**(1)**:** 111-123.

65. Balendra R, Isaacs AM. C9orf72-mediated ALS and FTD: multiple pathways to disease. *Nat Rev Neurol* 2018; **14**(9)**:** 544-558.

66. Han M, Liu Z, Xu Y, Liu X, Wang D, Li F *et al.* Abnormality of m6A mRNA Methylation Is Involved in Alzheimer's Disease. *Front Neurosci* 2020; **14:** 98.

67. Segklia A, Seuntjens E, Elkouris M, Tsalavos S, Stappers E, Mitsiadis TA *et al.* Bmp7 regulates the survival, proliferation, and neurogenic properties of neural progenitor cells during corticogenesis in the mouse. *PLoS One* 2012; **7**(3)**:** e34088.

68. Tochigi M, Kato C, Otowa T, Hibino H, Marui T, Ohtani T *et al.* Association between corticotropin-releasing hormone receptor 2 (CRHR2) gene polymorphism and personality traits. *Psychiatry Clin Neurosci* 2006; **60**(4)**:** 524-526.

69. Amin N, Hottenga JJ, Hansell NK, Janssens AC, de Moor MH, Madden PA *et al.* Refining genome-wide linkage intervals using a meta-analysis of genome-wide association studies identifies loci influencing personality dimensions. *Eur J Hum Genet* 2013; **21**(8)**:** 876-882.

70. Hedtke V, Bakovic M. Choline transport for phospholipid synthesis: An emerging role of choline transporter-like protein 1. *Exp Biol Med (Maywood)* 2019; **244**(8)**:** 655-662.
