# Supplementary material for: Gene expression networks regulated by human personality
Source: Mol Psychiatry. 2024 Mar 4;29(7):2241–60. doi: 10.1038/s41380-024-02484-x (PMC11408262; doi:10.1038/s41380-024-02484-x)
Supplement: Supplementary file 2 — Supplementary Figure S1 [file 41380_2024_2484_MOESM2_ESM.pdf]

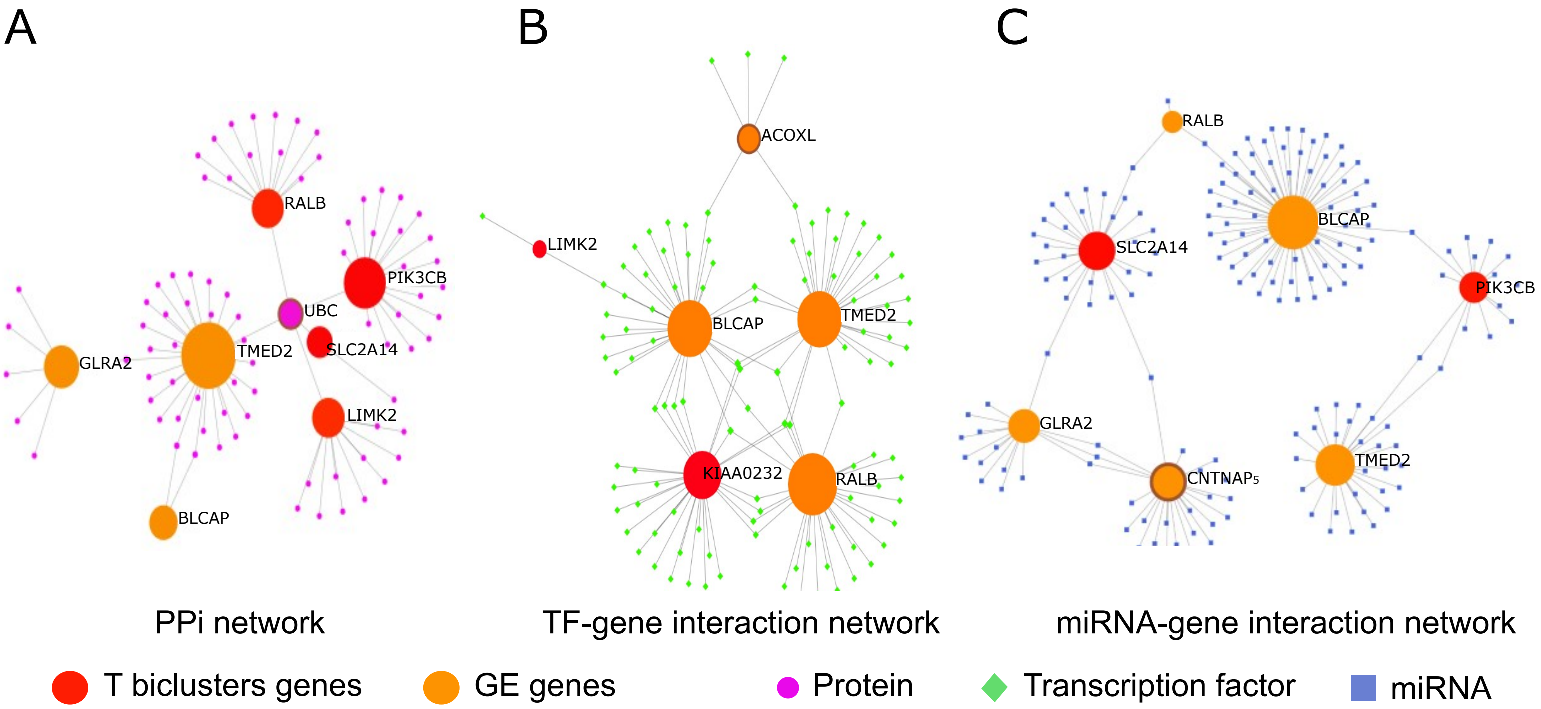
A
B
C
RALB
PIK3CB
UBC
SLC2A14
GLRA2
TMED2
LIMK2
BLCAP
ACOXL
LIMK2
BLCAP
TMED2
KIAA0232
RALB
RALB
BLCAP
SLC2A14
PIK3CB
GLRA2
CNTNAP5
TMED2
PPi network
TF-gene interaction network
miRNA-gene interaction network
T biclusters genes
GE genes
Protein
Transcription factor
miRNA
