# Supplementary figures and images for: Gene expression networks regulated by human personality
Source: Mol Psychiatry. 2024 Mar 4;29(7):2241–60. doi: 10.1038/s41380-024-02484-x (PMC11408262; doi:10.1038/s41380-024-02484-x)

A

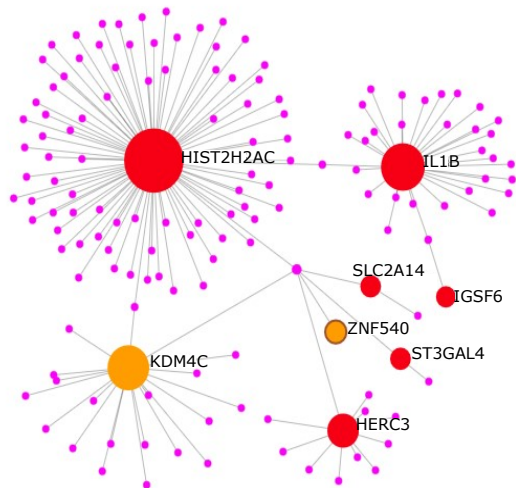

PPI network

B

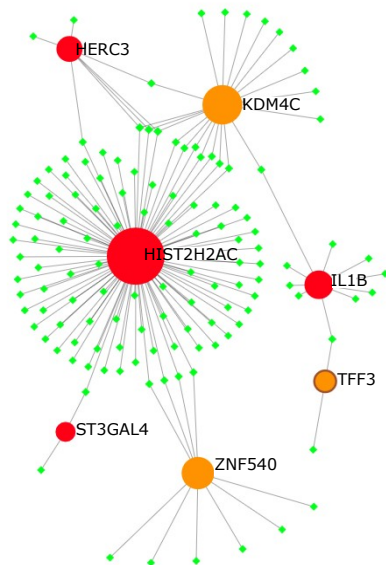

TF-gene interaction network

C

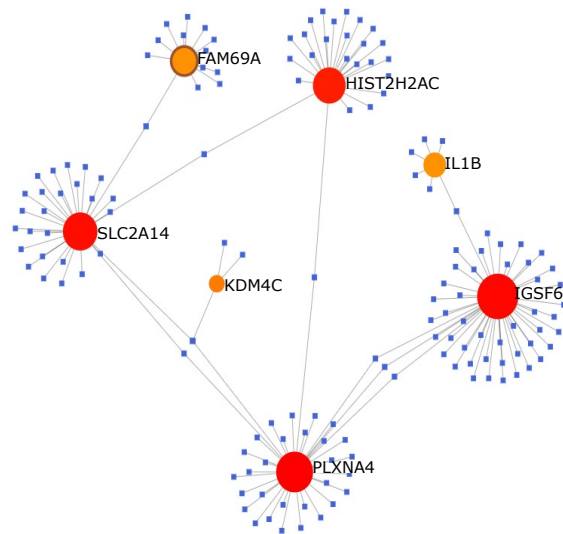

miRNA-gene interaction network

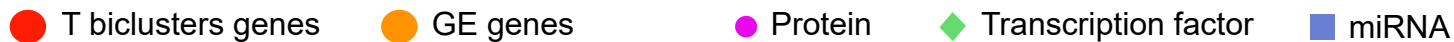

Supplement: Supplementary file 3 — Supplementary Figure S2 [file 41380_2024_2484_MOESM3_ESM.pdf]

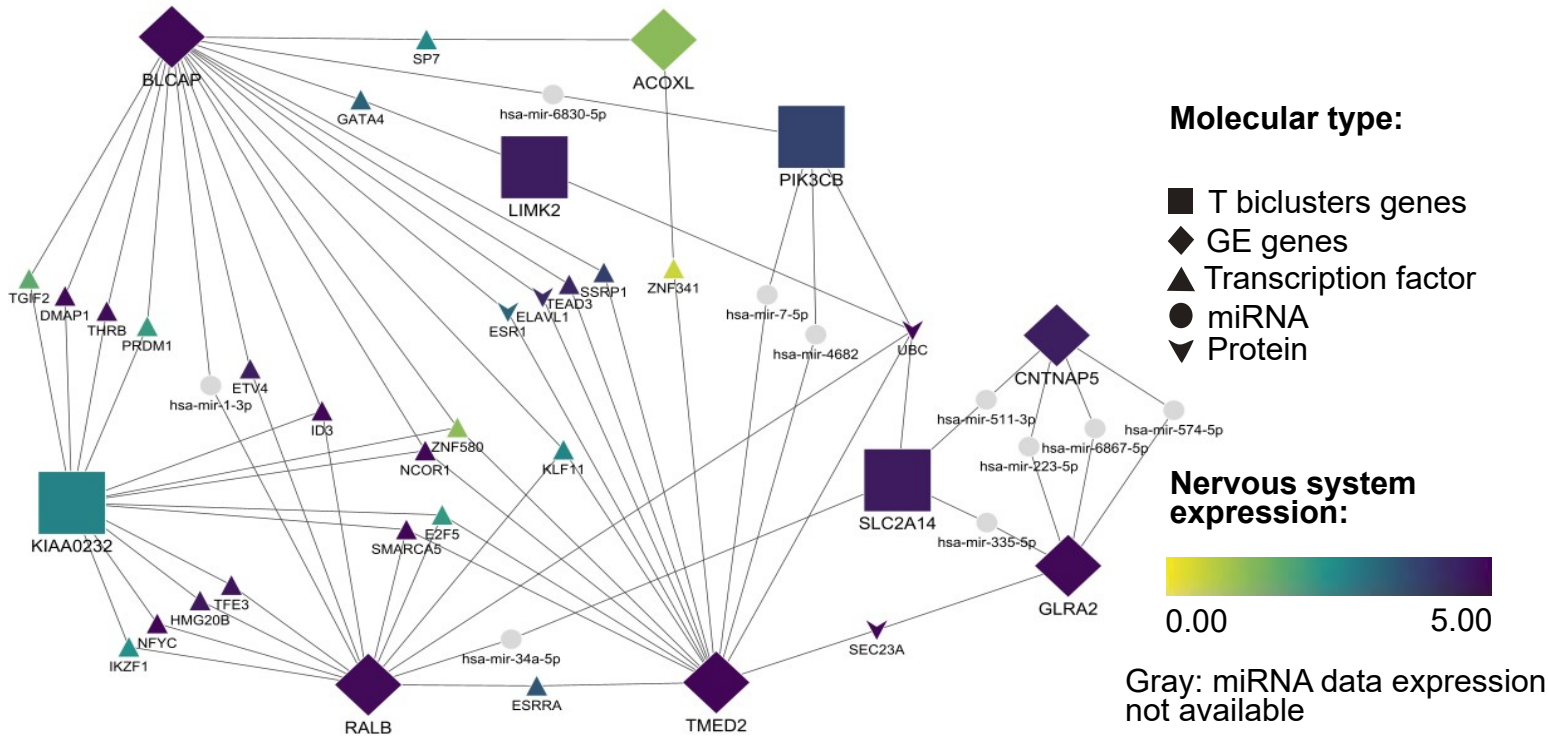

Supplement: Supplementary file 4 — Supplementary Figure S3 [file 41380_2024_2484_MOESM4_ESM.pdf]

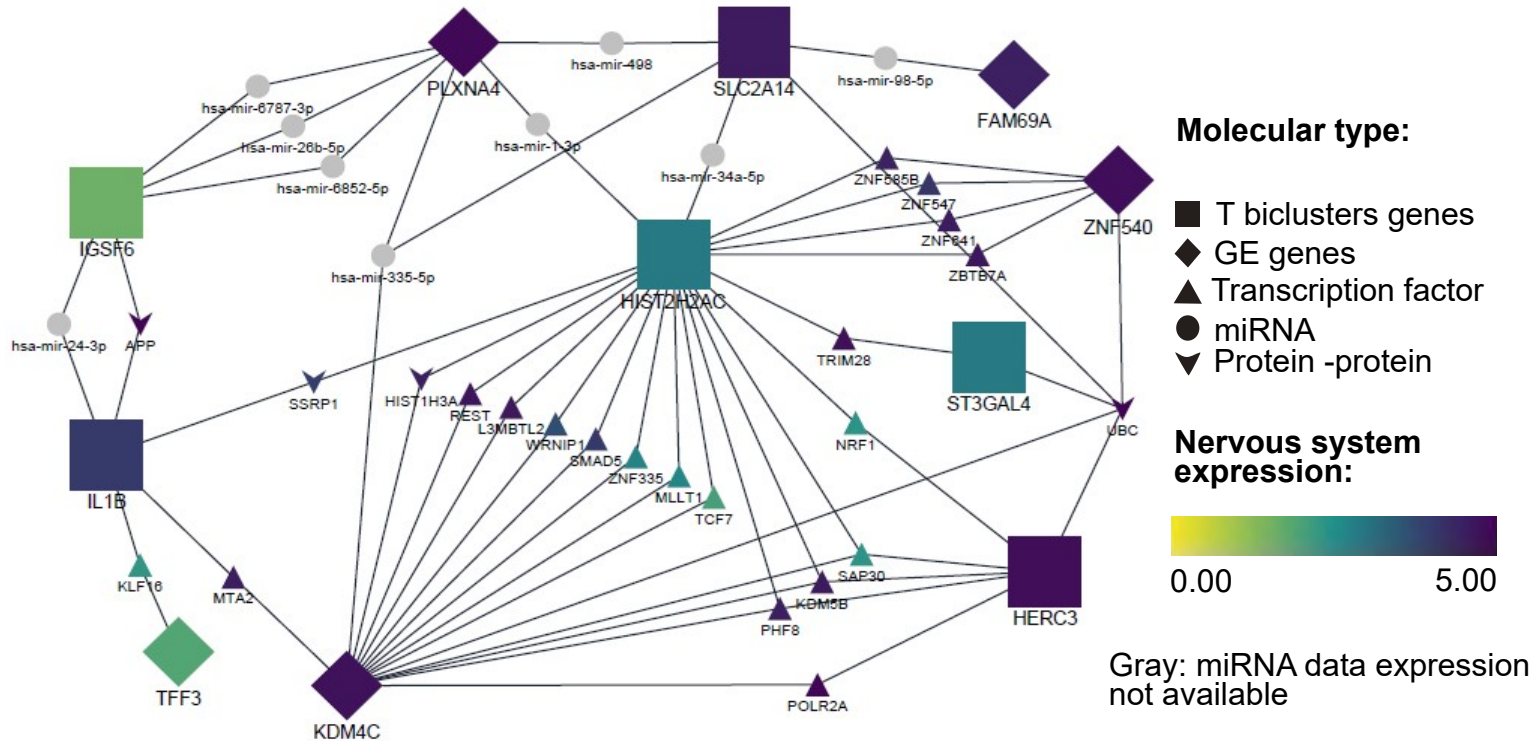

Supplement: Supplementary file 5 — Supplementary Figure S4 [file 41380_2024_2484_MOESM5_ESM.pdf]

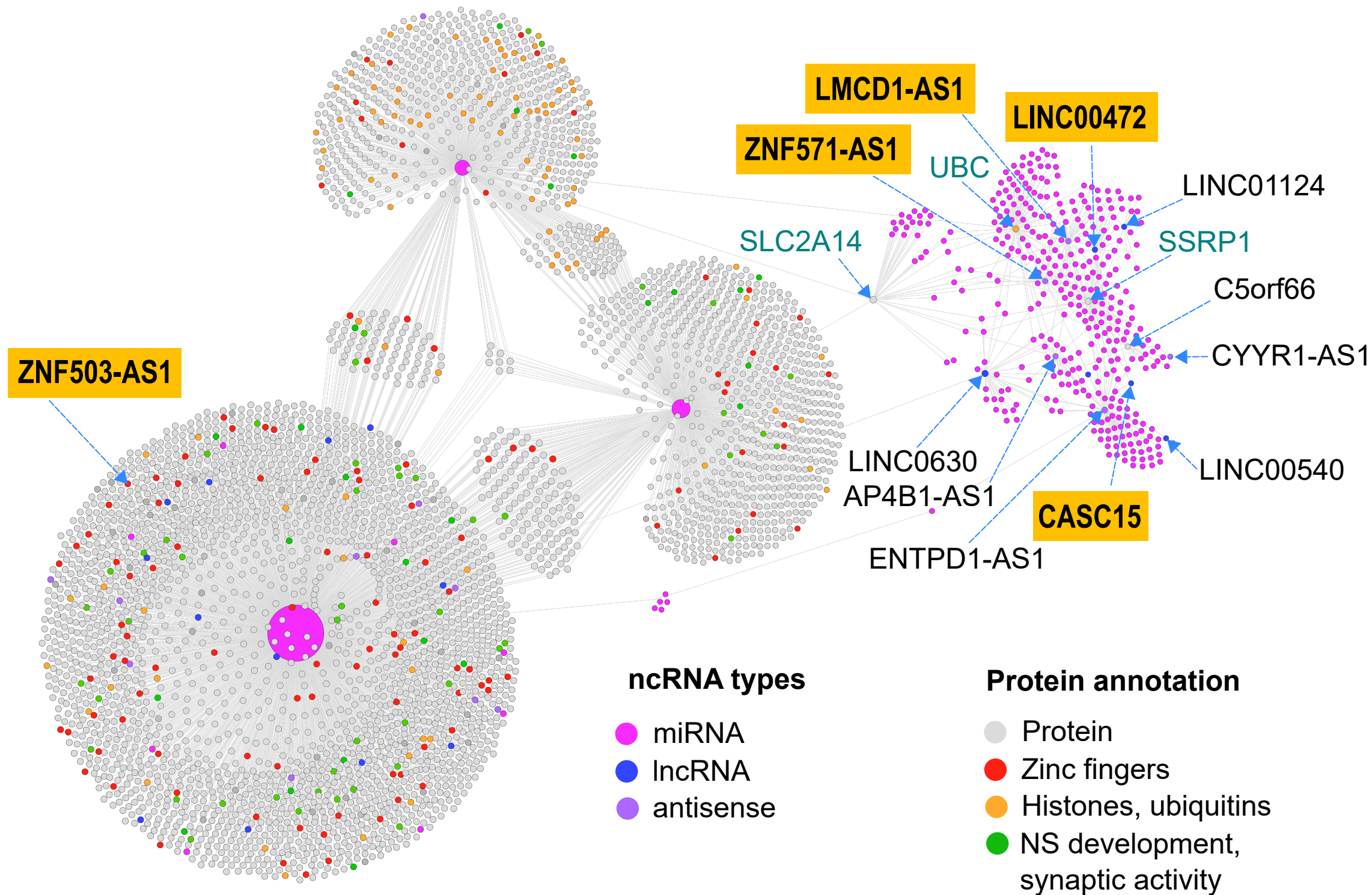

Supplement: Supplementary file 6 — Supplementary Figure S5 [file 41380_2024_2484_MOESM6_ESM.pdf]

TARGET

hsa-mir-1-3p  
hsa-mir-335-5p  
hsa-mir-34a-5p

95% < & >= 90%

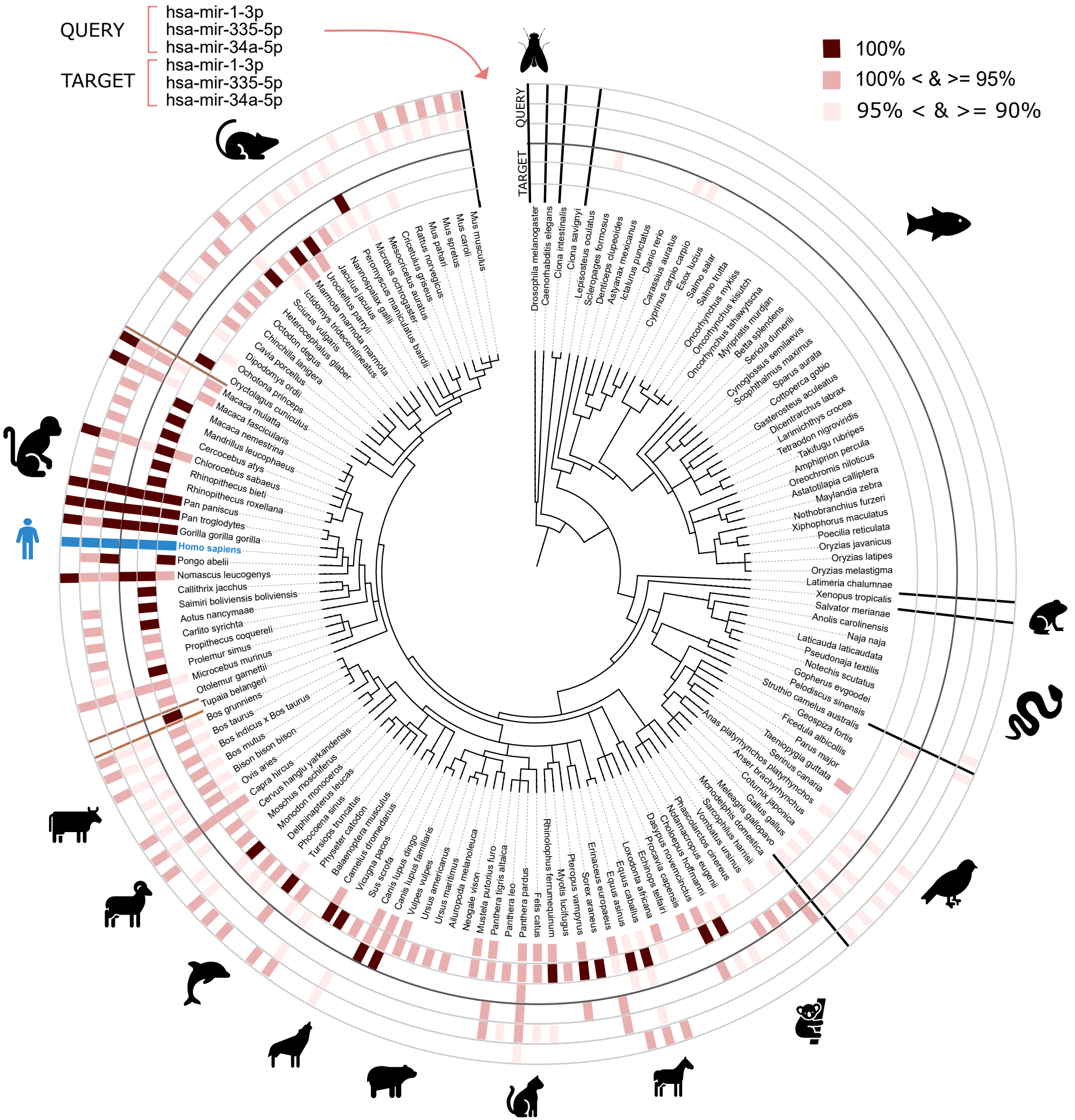

Supplement: Supplementary file 7 — Supplementary Figure S6 [file 41380_2024_2484_MOESM7_ESM.pdf]
